# The respiratory syncytial virus M2-2 protein is targeted for proteasome degradation and inhibits translation and stress granules assembly

**DOI:** 10.1371/journal.pone.0289100

**Published:** 2023-07-25

**Authors:** Orlando Bonito Scudero, Verônica Feijoli Santiago, Giuseppe Palmisano, Fernando Moreira Simabuco, Armando Morais Ventura

**Affiliations:** 1 Department of Microbiology, Institute of Biomedical Sciences, University of São Paulo, São Paulo, Brazil; 2 Department of Parasitology, Institute of Biomedical Sciences, University of São Paulo, São Paulo, Brazil; 3 Multidisciplinary Laboratory of Food and Health, School of Applied Sciences, University of Campinas, Limeira, São Paulo, Brazil; 4 Department of Biochemistry, Federal University of São Paulo, São Paulo, SP, Brazil; CRCL: Centre de Recherche en Cancerologie de Lyon, FRANCE

## Abstract

The M2-2 protein from the respiratory syncytial virus (RSV) is a 10 kDa protein expressed by the second ORF of the viral gene M2. During infection, M2-2 has been described as the polymerase cofactor responsible for promoting genome replication, which occurs by the induction of changes in interactions between the polymerase and other viral proteins at early stages of infection. Despite its well-explored role in the regulation of the polymerase activity, little has been made to investigate the relationship of M2-2 with cellular proteins. A previous report showed poor recruitment of M2-2 to viral structures, with the protein being mainly localized to the nucleus and cytoplasmic granules. To unravel which other functions M2-2 exerts during infection, we performed proteomic analysis of co-immunoprecipitated cellular partners, identifying enrichment of proteins involved with regulation of translation, protein folding and mRNA splicing. In approaches based on these data, we found that M2-2 expression downregulates eiF2α phosphorylation and inhibits both translation and stress granules assembly. Finally, we also verified that M2-2 is targeted for proteasome degradation, being localized to granules composed of defective ribosomal products at the cytoplasm. These results suggest that besides its functions in the replicative complex, M2-2 may exert additional functions to contribute to successful RSV infection.

## Introduction

The respiratory syncytial virus (RSV) is an enveloped, single-stranded, negative RNA virus belonging to the order *Mononegavirales* family *Pneumoviridae* [1]. It is one of the major causes of lower tract respiratory disorders, being mainly responsible for infections in newborns, infants, elderly, and immunocompromised individuals. Despite its importance as a pathogen, currently there are no vaccines or effective drugs available against the virus, turning its replicative cycle into an interesting target for the discovery of new antiviral agents [2, 3].

RSV infection starts with the fusion of the viral envelope to the cell membrane and the release of its ribonucleoprotein complex into the cytoplasm. The last comprises the viral nucleocapsid, the RNA-dependent RNA polymerase (RdRp) and its cofactor for transcription. The nucleocapsid is composed by the viral genome associated with the nucleoprotein N, which works as a template for the polymerase while it keeps the genomic RNA hidden [4]. The RdRp has two main components, the phosphoprotein P, a tetrameric protein able to interact with the nucleocapsid and the large protein L, responsible for the catalytic activity of the polymerase [5, 6]. The last performs both transcription and replication activities, which are coordinated by cofactors transcribed from the two open reading frames (ORFs) of the gene M2. The first ORF translates for the M2-1 protein, a regulator of transcription and structural component of the virion, while the second ORF translates for the M2-2 protein, thought to direct the polymerase activity for replication [7–9].

Once the ribonucleoprotein complex gets into the cytoplasm, it starts rounds of transcription to produce viral proteins. At few hours post infection, cytoplasmic inclusion bodies (IBs) are assembled, a process triggered by the proteins N and P, able to phase separate when co-expressed [10, 11]. These structures work as viral factories for genome transcription and replication, processes highly regulated by M2-1 and M2-2. M2-1 is essential for viral transcription, and its role on the synthesis and processing of viral mRNAs has been broadly explored [12, 13]. On the other hand, M2-2 function was firstly deduced from the phenotype produced by a mutant virus lacking its ORF. Infection by this mutant showed enhanced mRNA production with delayed accumulation of viral genome, suggesting a role in switching the polymerase activity for replication [7, 14]. This observation was recently confirmed by Blanchard and colleagues, showing that M2-2 expression can rearrange the interactions between L and other IB components [15].

In addition to its exerted functions in replication, viral proteins are thought to hijack cellular components to assist viral infection or to impair cellular metabolism and host defenses. In this context, RSV proteins have been shown to interfere with many cellular processes. Non-structural proteins NS1 and NS2 prevent IFN I induction, and NS1 is also capable to downregulate the expression of interferon-stimulated genes in the nucleus [16, 17]. In the same way, the matrix protein M also localizes to the nucleus and impairs the expression of genes related to mitochondrial metabolism [18]. On the cytoplasm, N and P hijack cellular chaperones to aid viral replication and impair host defenses by sequestering and keeping innate immune effectors inside of IBs [19, 20]. Accordingly, M2-1 also recruits cellular proteins related to mRNA maintenance to support the synthesis and processing of viral transcripts [13, 21].

While the functions of many RSV proteins in hijacking the cellular machinery have been extensively studied, little has been done to investigate the relationship of M2-2 with cellular components. Previous data on Vero E6 cells showed that M2-2 is poorly localized to IBs, and accumulates in cytoplasmic granules and nucleus, suggesting that it may exert additional functions in the course of infection [15]. To look at this question, in this work we investigated the relationship between M2-2 and the cellular machinery. To circumvent the lack of antibodies against M2-2, genes fused to FLAG and EGFP sequences were synthesized to express and detect the protein in eukaryotic cells. Expressing FLAG-tagged M2-2, we identified by co-immunoprecipitation assay coupled to mass spectrometry analysis a high correlation of M2-2 with ribosomal components, chaperones, splicing factors, and proteasome subunits. Looking for an association between proteomic data and M2-2 cytoplasmic granules, we found that M2-2 expression downregulates eiF2α phosphorylation and prevents stress granules assembly. Additionally, we also verified the ability of M2-2 in regulating translation, being able to inhibit both 5’ cap and IRES-dependent translations. Finally, we described the recruitment of M2-2 to cytoplasmic granules composed of defective ribosomal products (DRiPs) and showed that the protein is targeted for proteasome degradation, implying it may be involved with translation elongation as well. These data reveal new undescribed functions for M2-2 and suggest it as a multitask protein rather than just a polymerase cofactor.

## Materials and methods

### Cells and virus

HEK293T, HEp-2, A549 and Vero E6 cells were grown at 37°C in Dulbecco’s Modified Eagle Medium (DMEM) supplemented with 10% (v/v) fetal bovine serum (FBS), gentamicin (10 μg/mL) and tylosin (8 μg/mL). Cells were routinely tested for mycoplasma contamination. RSV strain A2 was amplified in HEp-2 cells at 37°C and after two passages, cells were scraped, resuspended in PBS containing 10 mM EDTA (pH 8.0) and Dounce homogenized. Lysates were then centrifuged for precipitation of cell debris (3,250 x g for 20 min at 4°C), and NT buffer (150 mM NaCl, 50 mM Tris–HCl, pH 7.5) containing 50% polyethylene glycol 6000 (w/v) was added to supernatants to a final concentration of 10%. Lysates were incubated for 90 min under gentle agitation (4°C) and then centrifuged (3,250 x g for 20 min at 4°C). Pellets containing purified virus were resuspended in NT buffer containing 15% sucrose (w/v) 1 mM EDTA and stored at -80°C. Plaque assays for stock titration were performed at 37°C in HEp-2 cells, using carboxymethylcellulose (Sigma) as overlay.

### Plasmids

Optimized genes for expression of FLAG-M2-2, EGFP-M2-2, FLAG-YB-1 and EGFP-YB-1 (S1 File) in human cells were synthesized and subcloned in the vector pcDNA3.1(+) at GeneArt (Thermo Fisher Scientific). FLAG-M2-2 gene was inserted between the NcoI and AflII restriction sites, while the other three genes were inserted between NheI and ApaI sites. The optimized gene for M2-1 (S1 File) expression was acquired at GeneArt (Thermo Fisher Scientific), subcloned downstream to FLAG in the vector pcDNA3.1-FLAG [22] (between the BamHI and EcoRI sites) and analyzed by sequencing. Optimization was performed utilizing amino acid sequences obtained on Uniprot (RSV strain A2 for viral genes and *Homo sapiens* for YB-1 protein), and the nucleotide sequence of the optimized genes is provided in the Supplementary Information. Additionally, pcDNA3.1-FLAG (empty vector) and pcDNA3.1-FLAG-EGFP were utilized as control vectors. The vector pLPL, used for the luciferase reporter assay, was previously described [23].

### Transfection and infection

Transfection was performed with Lipofectamine 3000 (Thermo Fisher Scientific) following manufacture’s recommendations. Briefly, cells were transfected with 0.3 μg of DNA for each cm2 (transfection surface area), using lipofectamine at the proportion 1:3 (DNA / Lipofectamine) in Opti-MEM (Thermo Fisher Scientific), and after 4 hours, medium was changed for DMEM 1% FBS. For infection, cells were incubated with RSV for 1h at 37°C in DMEM containing 1% FBS, and after adsorption, medium was changed. Transfection for expression of M2-2 plasmids in infected cells was performed 10 hours post-infection, as previously described [15]. For the luciferase reporter assay, cells were transfected 1 hpi. For all experiments, cells were plated the day before to an expected confluence of 70 to 80%.

### Reagents and treatments

ISRIB (SML0843 –Sigma) diluted in DMSO was used at concentrations of 200 nM and 500 nM, and treatments were performed after a change of transfection media at 4 hours post transfection (hpt) until cell lysis or fixation. Arsenite (S7400 –Sigma) diluted in sterile water was used at concentrations of 0.5 mM for 30 min in HEK293T, HEp-2 and A549 cells, and 1 mM for 1h in Vero E6 cells. Puromycin was used at the final concentration of 10 μg/mL, and was incubated for 10 min for SUnSET assays, using co-treatment with cycloheximide (100 μg/mL) as a negative control [24]. For immunofluorescence, cells were incubated with puromycin (10 μg/mL) and MG132 (Abcam) (5 μM) for 2h. Additionally, MG132 treatments were performed for 2h (immunofluorescence) and overnight (4 hpt until cell lysis–for western blot) at 5 μM. The HSP70 inhibitor VER-155008 (Sigma) was used at concentrations of 2.5 μM and 5 μM, with similar treatments to ISRIB.

### Antibodies

The following antibodies were used. Anti-FLAG mouse (F1804 –Sigma, 1:200 IF, 1:2000 WB), anti-FLAG rabbit (F7425 –Sigma, 1:200 IF, 1:2000 WB), anti-GFP rabbit (sc-8334 –Sta Cruz, 1:100 IF, 1:1000 WB), anti-N mouse (ab94806 –Abcam, 1:200 IF), anti-P mouse (ab94965 –Abcam, 1:2000 WB), anti-G3BP1 rabbit (#61559 –Cell Signaling, 1:200 IF), anti-Calreticulin rabbit (ab2907 –Abcam, 1:100 IF), anti-Phospho-eiF2α rabbit (#3398 –Cell Signaling, 1:1000 WB), anti-eiF2α rabbit (#5324 –Cell Signaling, 1:2000 WB), anti-β-tubulin rabbit (ab6046 –Abcam, 1:5000 WB), anti-PABP mouse (sc-32318 –Sta Cruz, 1:10 IF), anti-HSP70 mouse (H5147 –Sigma, 1:2000 WB), anti-BiP rabbit (#3183 –Cell Signaling, 1:1000 WB), anti-Ubiquitin rabbit (#3369 –Cell Signaling, 1:1000 WB), anti-Puromycin mouse (MABE343 –Sigma, 1:200 IF, 1:5000 WB), anti-GAPDH rabbit (ab9485 –Abcam, 1:2000 WB), anti-S6 rabbit (#2217—Cell Signaling, 1:2000 WB), anti-Phospho-4E-BP1 rabbit (#2855 –Cell Signaling, 1:1000 WB), anti-4E-BP1 rabbit (#9452 –Cell Signaling, 1:1000 WB), anti-Mouse HRP (ab97023 –Abcam, 1:5000 WB), anti-Rabbit HRP (ab97051 –Abcam, 1:5000 WB), anti-Mouse Alexa Fluor 594 (A11032 –Invitrogen, 1:1000 IF), anti-Rabbit Alexa Fluor 488 (A11034 –Invitrogen, 1:1000 IF), anti-Rabbit Alexa Fluor 594 (A11012 –Invitrogen, 1:500 IF).

### Co-immunoprecipitation

HEK293T cells at 70% confluence were transfected with FLAG-M2-2 or empty vector and after 48h cells were rinsed and lysed for 15 min on ice (50 mM Tris-HCl pH 7.5, 150 mM NaCl, 1 mM EDTA, 1% Triton X-100 (v/v), protease and phosphatase inhibitors (Sigma). Lysates were then centrifuged (5,000 x g for 10 min at 4°C) and supernatants incubated with FLAG-beads (Sigma) under gentle agitation (overnight at 4°C), saving a small sample for posterior analysis. Next steps were performed following manufacture’s recommendations. Co-immunoprecipitated proteins were eluted for 1h at 4°C with 3X FLAG peptide (Sigma) in TBS (pH 7.4). Samples were then quantified by BCA assay and analyzed by western blot.

### Sample preparation and mass spectrometry analysis

Eluted proteins from co-IPs were submitted to trypsin digestion. Briefly, proteins were reduced with Dithiothreitol (DTT) to a final concentration of 10 mM, incubated for 45 min at 56°C and subsequently alkylated with iodoacetamide (IAA) for 30 min at room temperature in the dark (final concentration 40 mM). Remaining IAA was quenched by DTT (final concentration 5 mM), and then Sequencing Grade Modified Trypsin (Promega) was added at the proportion 1:50 trypsin:protein ratio (w/w) following overnight incubation at 37°C. The samples were acidified adding trifluoroacetic acid (TFA) to a final concentration of 1% and digested peptides were desalted using C18 microcolumns (Thermo Fischer Scientific). Co-IP digested peptides were analyzed by Nano-HPLC-ESI-QUAD-TOF using a maXis 3G Bruker Daltonics (Central Analítica–IQ–USP). In brief, peptides were separated at a flow of 200 nl/min on a nanoAcquity UPLC® 1.8μm HSS T3 (75 μm x 200 mm) by reversed-phase chromatography, which was operated on a NanoAcquity Waters (Waters). The mobile phase was water/0.1% Formic Acid (A) and ACN/0.1% Formic Acid (B) during 240 minutes. The gradient was 2–30% phase B for 210 minutes, 30–85% B for 15 minutes, and 85–2% B for 15 minutes. The NanoAcquity Waters was coupled into a MAXIS 3G mass spectrometer (Bruker Daltonics) operating in positive ion mode. The mass spectrometer acquired a full MS scan at 60,000 full width half maximum (FWHM) resolution with a 350–2200 Da mass range. Top 10 most intense ions were selected from MS for Collision Induced Dissociation (CID) fragmentation (normalized collision energy: 6V). Exclusion time 120 seconds and 2 to 5 change range.

### MS/MS counting and label-free quantification

Bruker Q-TOF files were imported to MaxQuant version 1.6.17.0 for protein identification and quantification. For protein identification in MaxQuant, the database search engine Andromeda was used against Uniprot *Homo sapiens* (20,370 entries release). The following parameters were used: carbamidomethylation of cysteine (57.021464 Da) as a fixed modification, oxidation of methionine (15.994915 Da) and N-terminal acetylation protein (42.010565 Da) were selected as variable modifications. Enzyme specificity was set to full trypsin with maximum of two missed cleavages. The minimum peptide length was set to 7 amino acids. For label-free quantification, it was used “match between runs” feature in MaxQuant, which is able to identify the transfer between the samples based on the retention time and accurate mass, with a 0.7-minute match time window and 20 minutes’ alignment time window. Normalized MS/MS count was used as main parameter to further analyses. The mass spectrometry proteomics data is available at the ProteomeXchange Consortium (https://www.ebi.ac.uk/pride) via the PRIDE [25] partner repository with the dataset identifier PXD038402.

### Protein network and enrichment analysis

After the identification of peptides by normalized MS/MS count, proteins were selected as potential M2-2 binding partners by the following steps: (1) selection of enriched peptides (Fold Change > 1.5) or unique proteins for each independent experiment, excluding proteins present in negative controls, (2) selection of proteins identified in at least two out of three independent experiments. From this filtering, 72 proteins were selected as potential M2-2 interactors. These proteins were analyzed on STRING, for the generation of a functional protein-protein association network. Linking between network nodes was set for confidence, with minimum interaction score of 0.900. Analysis of enriched GO terms for Biological Process was performed on BiNGO (Cytoscape 3.9.1.), looking for overrepresented terms. Hypergeometric test was used for statistical analysis, with Benjamini-Hochberg False Discovery Rate correction, and significance set as 0.01.

### Western blot

For protein analysis by western blot, cells were lysed in RIPA buffer (50 mM Tris-HCl pH 8.0, 150 mM NaCl, 1% Triton X-100, 0.5% sodium deoxycholate, 0.1% SDS) and quantified using the QuantiPro BCA Assay Kit (Sigma). Samples were then boiled in Laemmli Buffer for 10 min and submitted to SDS-PAGE, followed by transfer to 0.2 μm nitrocellulose membrane (Sigma) and blocking with 5% BSA (w/v) or 5% non-fat milk diluted in PBS-T (PBS 0.1% Tween 20). Primary and secondary antibodies were incubated overnight at 4°C and 1h at room temperature, respectively. After each incubation, membranes were rinsed three times for 5 min with PBS-T. HRP detection was performed with Clarity Western ECL Substrate (Bio-Rad). For protein normalization, membranes were stripped for 30 min at room temperature with stripping buffer (0.2 M glycine, 0.1% SDS, 1% Tween 20, pH 2.2) and washed three times with PBS-T before the next probing. Western blot raw images are available on supporting files (S1 Raw images).

### Immunofluorescence staining

For immunofluorescence assays, cells were grown in glass coverslips at 70% confluence, and then submitted to transfection, infection, and treatments. 24h post treatments, cells were fixed with PBS containing 4% paraformaldehyde (w/v) for 15 min at 4°C, permeabilized with PBS 0.2% Triton X-100 for 15 min at 37°C and blocked with blocking solution (PBS, 0.1% Tween 20, 5% BSA) for 1h at 37°C. Primary and secondary antibodies were incubated in blocking solution overnight at 4°C and for 1h at 37°C, respectively, washing cells with PBS-T for three times between each step. Nuclei were stained with 4’,6-diamidino-2-phenylindole (DAPI) with secondary antibodies. Coverslips were mounted with Prolong Glass (Thermo Fisher Scientific), following curation for 24h in the dark at room temperature before imaging cells. For anti-calreticulin and anti-PABP staining, cells were fixed with ice-cold methanol for 10 min, following the same steps. Widefield microscopy was performed with a ZEISS Axio Vert.A1 microscope utilizing an objective Plan-NEOFLUAR 63x/NA 1.25 in oil immersion, and acquired images were analyzed on ImageJ (FIJI).

### Confocal microscopy

Confocal images were taken with a Zeiss LSM 780 NLO confocal microscope (Core Facility for Scientific Research–CEFAP-USP). Images were acquired with an objective α Plan-Apochromat 100x/NA 1.46 in oil immersion, using the following lasers: HeNe (543 nm), Argon (488 nm) and Diode (405 nm). Fluorescent signal was detected with a 32 channel GaAsP QUASAR detector with the following parameters: Alexa Fluor 594 (578–692 nm), Alexa Fluor 488 (491–587 nm), DAPI (412–491 nm). Pinhole was set to 1 airy unit, and z-stacks were taken with intervals of 290 nm. For deconvolution, theoretical PSF was generated, and images were processed using the DeconvolutionLab2 plugin [26] utilizing the Richardson-Lucy Total variation algorithm [27]. For all confocal immunofluorescences presented in this work, the images show a single z-plane. Images were contrast enhanced between the same experiments for better visualization.

### Reporter luciferase assay

HEK293T cells were co-transfected with pLPL (carrying Renilla and Firefly luciferases) [23] and FLAG-EGFP, FLAG-M2-2 or EGFP-M2-2. After 24h, cells were rinsed and lysed with passive lysis buffer (Promega). Luminescence assay was performed with the Dual-Glo Luciferase Assay System (Promega) following manufacture’s recommendations, and luciferase activity was measured on GloMax Discover (Promega). For the reporter assay in infected cells, HEK293T cells were infected for 1h, and then transfected for 4h, or alternatively, transfected for 4h and then infected for 1h. Lysis was performed after 24h.

### Cell fractionation in sucrose gradient

HEK293T cells were grown in T75 flasks at 50% confluence and then transfected with FLAG-EGFP, FLAG-M2-2 and EGFP-M2-2. After 24h, cells were incubated for 10 min with medium containing cycloheximide (100 μg/mL), following washing with ice-cold PBS. On ice, cells were then incubated with polysome extraction buffer (PEB—20 mM Tris-HCl pH 7.5, 100 mM NaCl, 5 mM MgCl2, 0.5% Triton X-100, 100 μg/mL cycloheximide, protease and RNAse inhibitors) for 10 min. Following incubation, cell lysates were centrifuged at 12,000 × g for 10 min at 4°C to pellet nuclei and debris. Supernatants were quantified by BCA assay and then 3 mg of protein extracts were loaded on the top of linear sucrose gradients (10–50%) prepared in polysome buffer (20 mM Tris-HCl pH 7.5, 100 mM NaCl, 5 mM MgCl2, 100 μg/mL cycloheximide). Samples were then centrifuged for 4h at 190,000 × g (4° C) with slow acceleration and brake (2) using a Beckman SW40Ti swinging bucket rotor. To analyze the generated gradient, fractions were collected and proteins were precipitated for western blot analysis [28]. In brief, 150 μl of each fraction were mixed with 600 μl of methanol and 150 μl of chloroform. 450 μl of water were then added to the mix, samples were vortexed and centrifuged at 20 000 × g for 5 min at 4°C. The aqueous layer was discarded and the pellet was washed twice with 650 μl of methanol, following centrifugation at 20 000 × g for 5 min at 4°C [29]. Pellets were dried and resuspended in Laemmli buffer for immunoblotting.

### Statistical analysis

Statistics was performed on GraphPad Prism 9.4.0, and details of statistical tests used, error bars and p-values are described in figure legends. Statistical tests were only performed in data obtained in independent biological replicates, represented on graphs by colored dots, where each color indicates paired values.

## Results

### Expression of FLAG and EGFP tagged M2-2 shows similar cellular distribution and partial localization to viral inclusion bodies

As mentioned above, the role of M2-2 on the modulation of the viral polymerase activity has already been shown by expressing a c-myc tagged M2-2 in Vero E6 cells co-infected with RSV [15]. Despite its known role as a polymerase cofactor for genome replication, M2-2 showed only partial recruitment to viral IBs, being mainly localized to nucleus and cytoplasmic granules, suggesting it may be involved in events other than regulating the polymerase activity.

**Fig 1:**
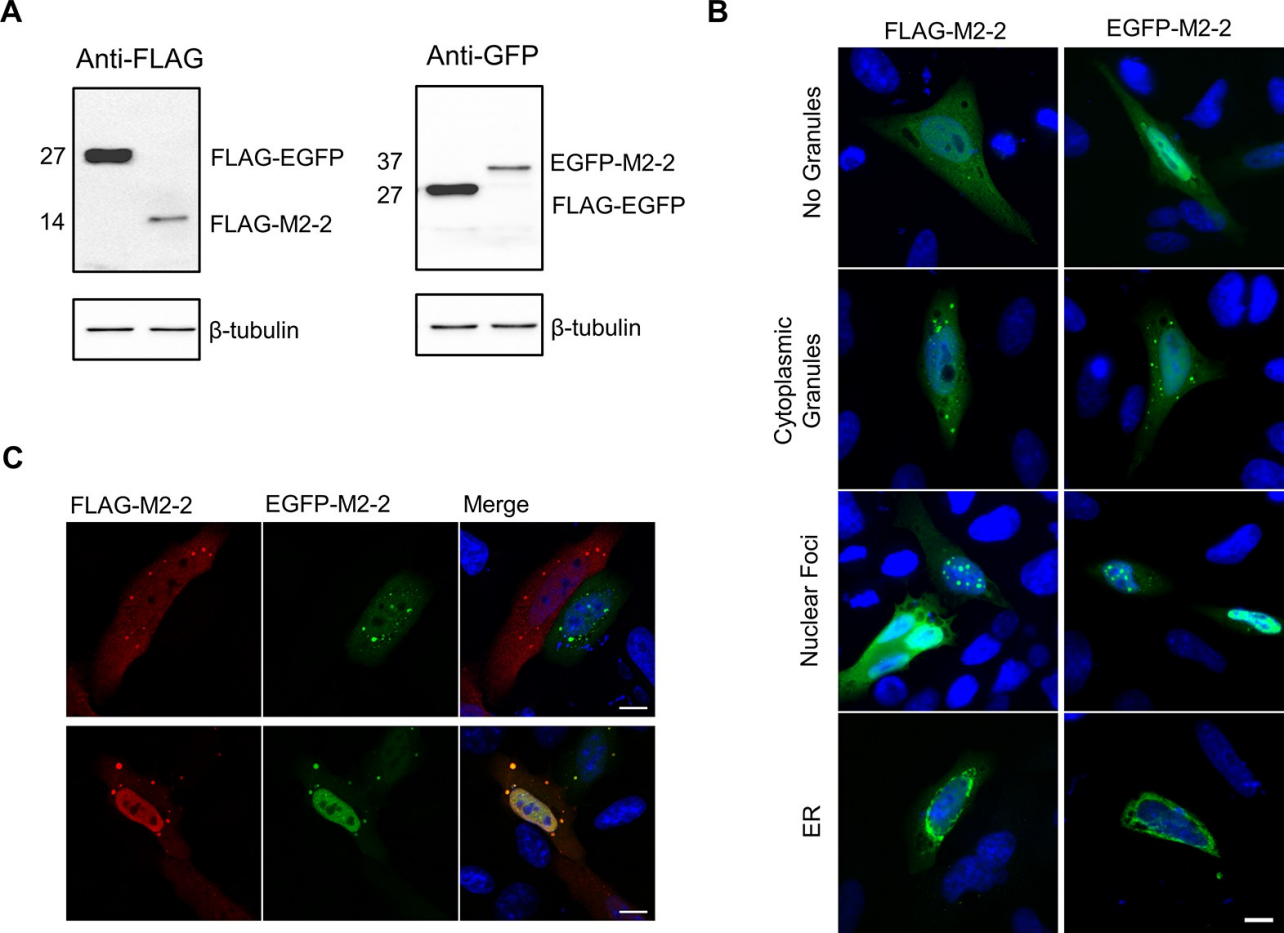
Characterization of recombinant M2-2 expression in human cell lines. (A) Detection of FLAG and EGFP-M2-2 expression in HEK293T cells at 24 hpt. FLAG-EGFP was used as a control for protein detection. Observed molecular weights are shown on the left. (B) Immunofluorescence of FLAG-M2-2 and EGFP-M2-2 expressed for 24h in HEp-2 cells. Panels show distinct distribution patterns observed for both proteins, as indicated on the left. (C) Confocal immunofluorescence showing co-expression of FLAG-M2-2 and EGFP-M2-2 in the same cell (second row) or expressed alone (first row). All images are representative of at least three independent experiments. Scale bars 10 μm.

To further explore this possibility, we synthesized two codon-optimized M2-2 genes, tagged by FLAG or EGFP at their amino terminus. Because M2-2 is a small 10 kDa protein, expressing it fused to EGFP could lead to its mislocalization, so we first evaluated the expression of both proteins in HEK293T cells, and their distribution in HEp-2 and Vero E6 cells (Fig 1A and 1B, S1 Fig). Similar to the previous report [15], both FLAG and EGFP-M2-2 presented nuclear and cytoplasmic localization in a tag-independent way. However, we also noticed cells where M2-2 assumed different patterns of distribution, presenting no cytoplasmic granules, showing nuclear foci or, at a minor amount, getting an ER-like shape. Despite their non-homogeneity, when co-expressed, FLAG and EGFP-M2-2 were recruited to the same sites, indicating that M2-2 may maintain its interactions regardless of the amino-terminal tags (Fig 1C).

**Fig 2:**
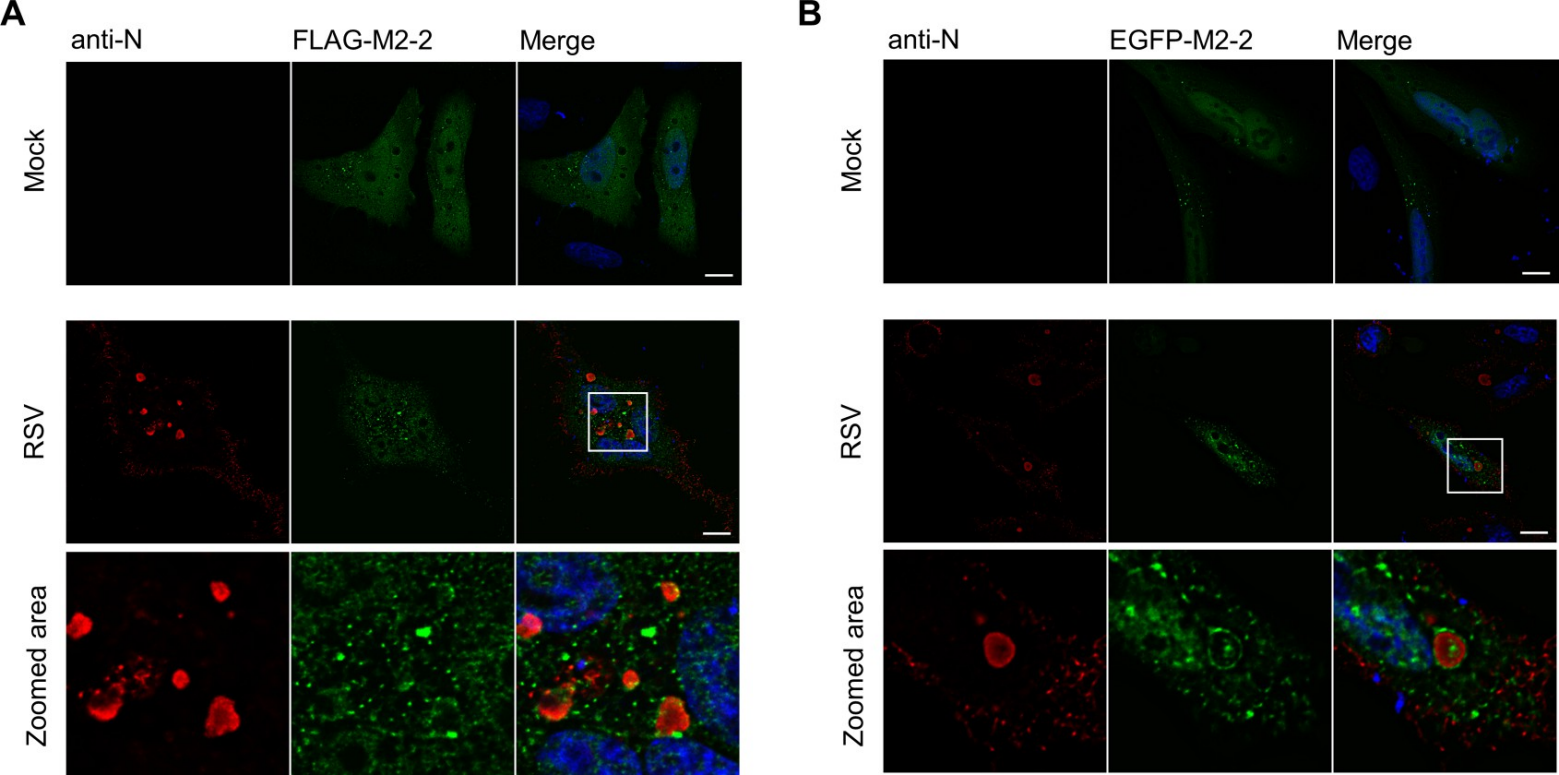
Expression of tagged M2-2 proteins in RSV infected human cells. (A) Expression of FLAG-M2-2 or EGFP-M2-2 (B) in mock or RSV infected HEp-2 cells. Images show the recruitment of M2-2 to the vicinity of viral IBs, stained with anti-N, as evidenced on zoomed areas. FLAG and EGFP-M2-2 localization to the nucleus and cytoplasmic granules is also observed. Raw confocal images were submitted to deconvolution as described in the methods section. All images are representative of at least three independent experiments. Scale bars 10 μm.

Finally, we also expressed FLAG and EGFP-M2-2 in infected HEp-2 cells, observing only a partial recruitment of both proteins to the vicinity of viral IBs (Fig 2A and 2B), reinforcing the previous report obtained in RSV co-infected Vero E6 cells [15]. It is important to emphasize that, during infection, M2-2 distribution maintains a pattern similar to that observed when the protein is expressed alone. Together, these data indicate the recruitment of M2-2 to IBs in a human cell line and point to its involvement with the cellular machinery in both nucleus and cytoplasm, either expressed alone or during infection. This makes way to investigate potential M2-2 cellular partners and to explore unidentified functions performed by the protein to enable successful infection.

### Proteomic analysis reveals translation and splicing machineries as potential interactors of M2-2

**Fig 3:**
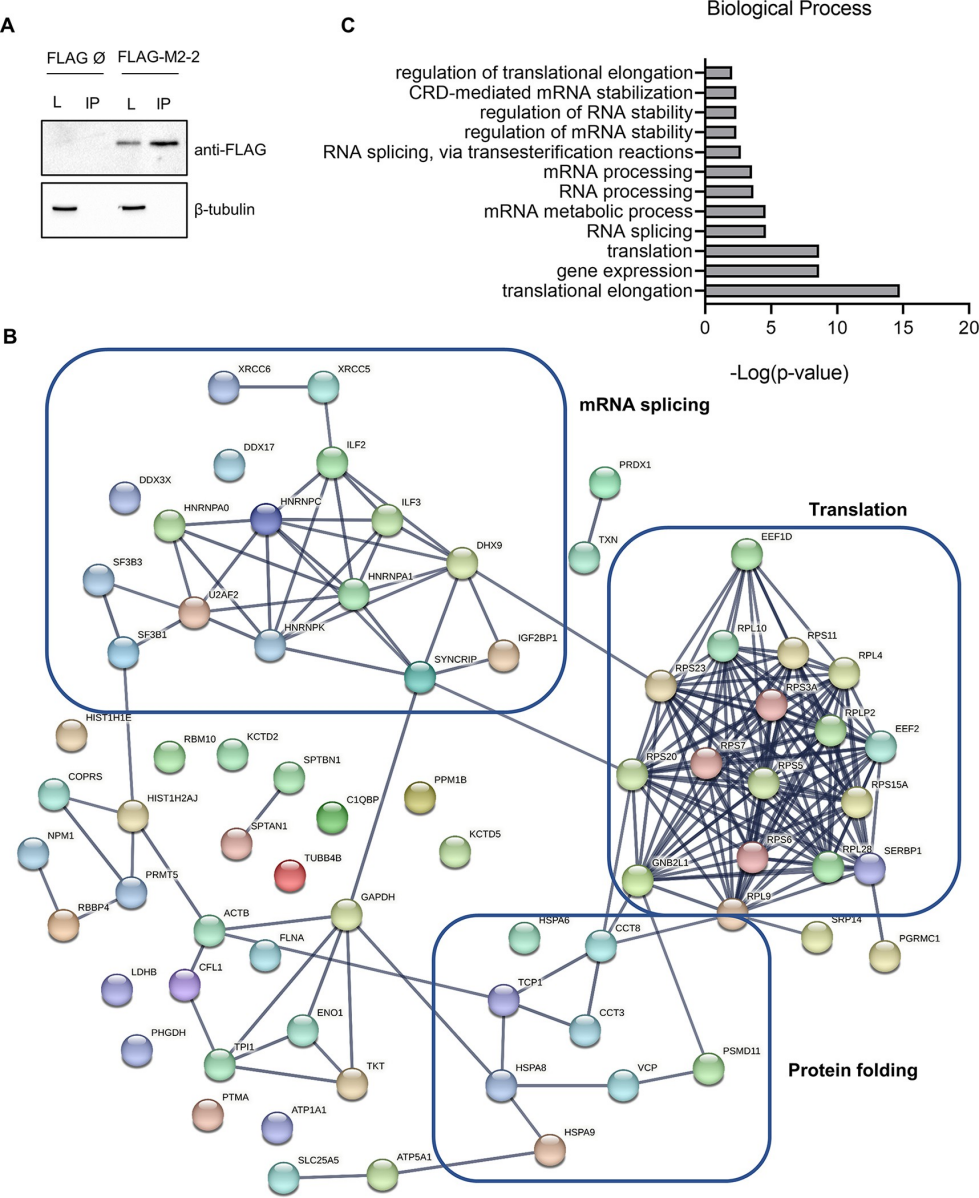
Proteomic analysis of M2-2 interactors shows enrichment of ribosome components, chaperones and splicing factors. (A) FLAG-M2-2 or empty vector were expressed in HEK293T cells for 48h and submitted to immunoprecipitation protocol. Western blot shows detection of FLAG-M2-2 in lysate (L) and eluted proteins (IP). Images are representative of three independent experiments sent for mass spectrometry analysis. (B) STRING network of the 72 proteins identified by mass spectrometry as potential interactors of M2-2. Lines connecting nodes indicate high-confidence interactions, as set during analysis. Protein names are indicated on nodes. Three main clusters are highlighted on the network, showing proteins related to translation, protein folding and mRNA splicing. (C) Graph showing enriched GO terms for Biological Process. Enrichment analysis for the identified proteins was performed on BinGO, as described in the methods section.

Aiming to identify potential M2-2 partners, we expressed FLAG-M2-2 or empty vector in HEK293T cells and performed immunoprecipitation (Fig 3A) followed by tryptic digestion and mass spectrometry analysis. Potential interactors identified in at least two of three independent experiments were selected for further analysis, after exclusion of proteins identified on negative control samples. Among the 72 proteins found (Fig 3B, S1 Table), we observed a surprising number of ribosomal proteins, splicing factors and chaperones as possible interactors. Accordingly, we identified enriched Gene Ontology (GO) terms for Biological Process associated to regulation of translation and translation elongation, and terms related to processing, stability and splicing of RNA and mRNA (Fig 3C).

These findings are endorsed by our previous observations, since M2-2 is localized to the nucleus, where mRNA synthesis and processing takes place, as well as at the cytoplasm, where it is mostly recruited to cellular granules rather than viral IBs during infection. Stress granules (SGs) and p-bodies are cytoplasmic granules known to be assembled by interactions between proteins and RNA and are described to be target of viral proteins to facilitate replication [30, 31]. Because the proteome of SGs harbor some of the ribosomal proteins, chaperones, and other RNA binding proteins [32] identified as potential partners of M2-2, we decided to investigate its relationship with these structures.

### M2-2 inhibits SGs assembly by keeping a low level of phosphorylated eiF2α

**Fig 4:**
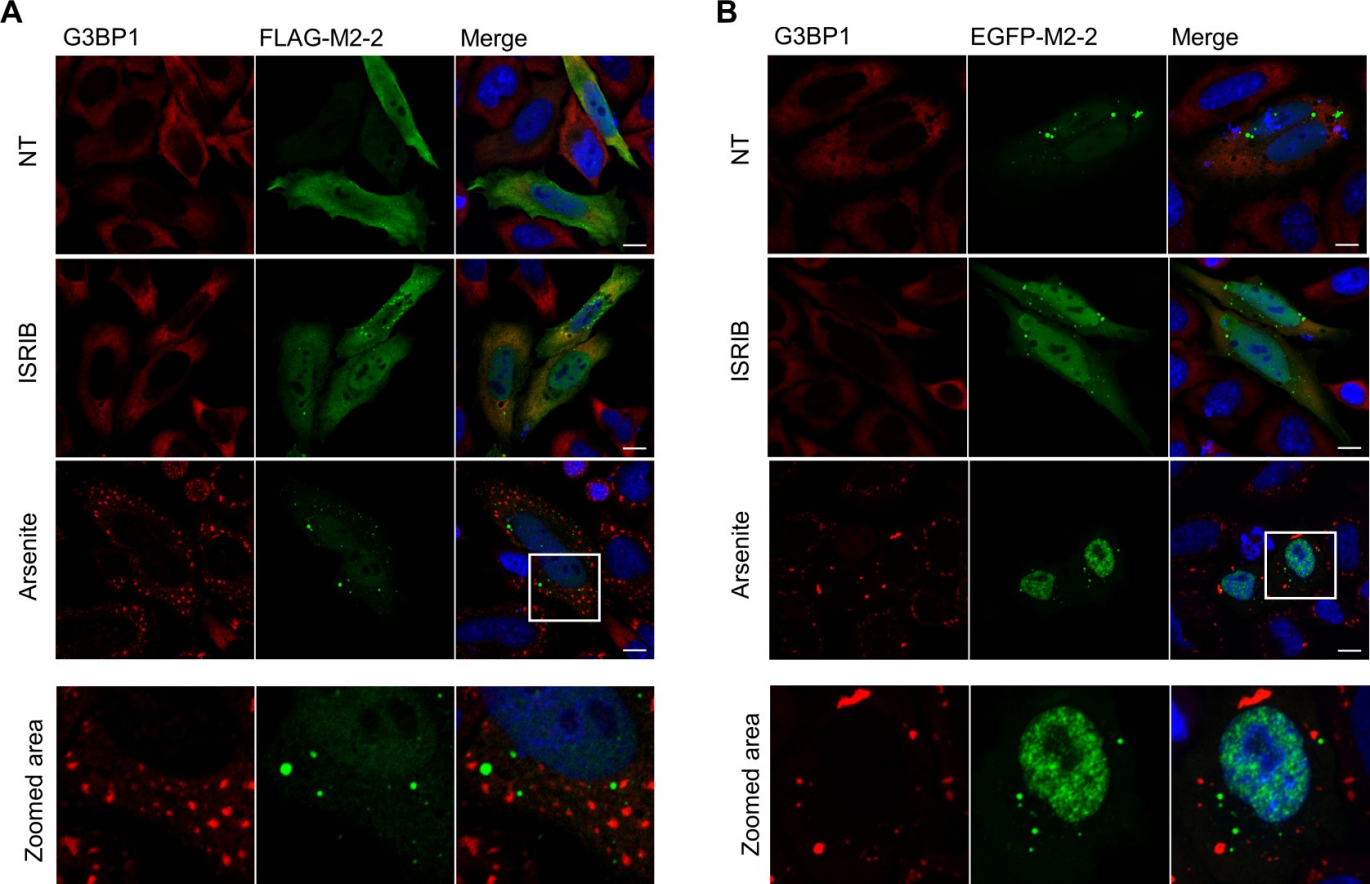
M2-2 does not colocalize with G3BP1 and stress granules. Expression of FLAG-M2-2 (A) and EGFP-M2-2 (B) in HEp-2 cells stained with anti-G3BP1. Cells were treated with 200 nM ISRIB (for 20h) or 0.5 mM arsenite for 30 min before fixation. Non-treated (NT) and ISRIB cells show diffuse G3BP1 distribution, while SGs can be observed under arsenite treatment. Distinction between M2-2 granules and SGs can be seen in zoomed areas. All images were taken with a Zeiss LSM-780-NLO microscope and are representative of three independent experiments. Scale bars 10 μm.

To identify if M2-2 is recruited to SGs, we expressed FLAG and EGFP-M2-2 in HEp-2 cells and performed immunofluorescence using as SG marker the protein G3BP1. As a negative control, cells were treated with the small molecule ISRIB (Integrated Stress Response Inhibitor), known to counteract the inhibitory effect of the phosphorylated eiF2α on the eiF2B complex, allowing the recycling of the eiF2 ternary complex and promoting disassembly of SGs [33, 34]. For induction of SGs, cells were treated with sodium arsenite, an inducer of oxidative stress. Fig 4A and 4B show that neither G3BP1 is recruited to M2-2 granules, nor ISRIB is able to promote their disassembly. Moreover, arsenite-induced SGs revealed to be distinct structures from the ones carrying M2-2 (Fig 4A and 4B—zoomed areas). Similar results were obtained during the co-expression of M2-2 and tagged YB-1 proteins, which are recruited for both SGs and p-bodies [35, 36], showing the absence of M2-2 in these granules as well (S2A and S2B Fig).

**Fig 5:**
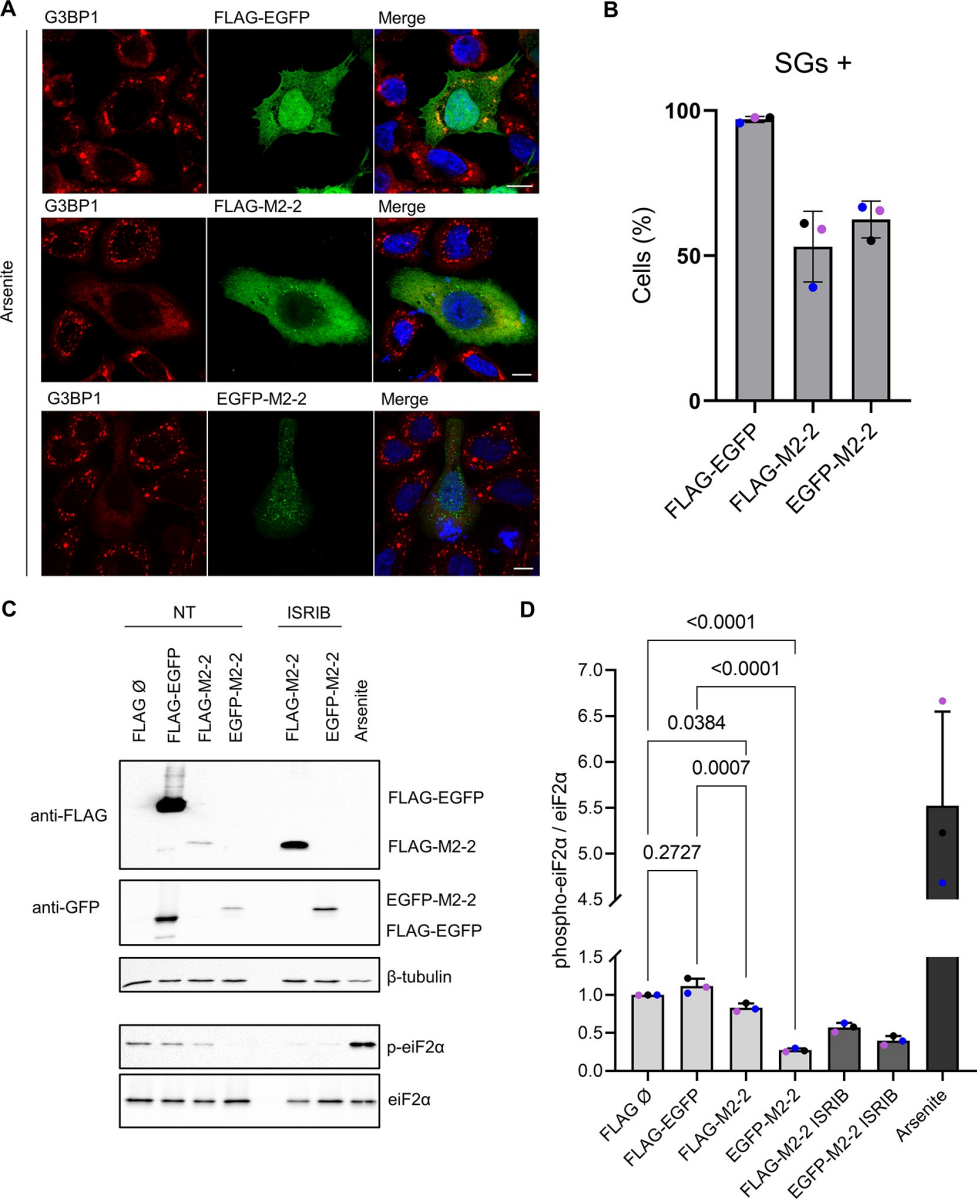
M2-2 expression inhibits SGs assembly and eiF2α phosphorylation. (A) Immunofluorescence of FLAG-EGFP, FLAG-M2-2 and EGFP-M2-2 in HEp-2 cells treated with arsenite (0.5 mM for 30 min). Inhibition of SGs assembly, stained by G3BP1, can be seen in cells expressing M2-2. (B) The graph presents the average proportion of cells shown in (A) positive for stress granules, showing reduction in SG assembly during the expression of M2-2. Roughly 30 cells were counted in each independent experiment, and colored dots indicate individual values for each experiment (n = 3). Error bars show standard deviation. (C) FLAG-M2-2 and EGFP-M2-2 or controls (FLAG-EGFP and empty vector) were transfected in HEK293T cells, and after 24h cells were lysed and analyzed by western blot. Treatments with ISRIB (200 nM) or arsenite (0.5 mM) were performed as previously described. Phosphorylation of eiF2α was quantified and submitted to one-way anova followed by Tukey’s multiple comparisons test (D). Graph presents mean values, with colored dots indicating values from independent replicates (n = 3). Error bars show standard deviation and p-values are shown in the graph.

Although no interaction with SGs has been observed, we did notice a portion of cells expressing M2-2 where arsenite treatment was not able to induce their assembly, while no effect was observed during the treatment of cells expressing the control FLAG-EGFP (Fig 5A). The quantification of cells positive for stress granules showed a reduction of roughly 40% in the proportion of cells presenting SGs in comparison with the control (Fig 5B). Comparable inhibitory effects were also observed for the protein PABP (S3A and S3B Fig), another SG component [32], or during M2-2 expression in A549 and Vero E6 cells (S3C and S3D Fig). Stress granules are assembled in response to eiF2α phosphorylation [31], therefore we expressed FLAG-M2-2 and EGFP-M2-2 or control vectors in HEK293T cells and evaluated the phosphorylation of eiF2α, using as negative control transfected cells treated with ISRIB (Fig 5C). Consistent with the above results, cells expressing M2-2 exhibited a marked reduction in the level of phosphorylated eiF2α compared to cells transfected with empty vector or FLAG-EGFP (Fig 5D). This implies that M2-2 may contribute to hamper host defenses coordinated by eiF2α phosphorylation during infection.

Previous reports already explored the relation between RSV infection and the activation of the integrated stress response mediated by eiF2α phosphorylation, showing that infection does not induce stress granules, and that RSV prevents eiF2α phosphorylation by the interaction between the viral protein N and the kinase PKR [37–39]. In our experiments, RSV infection did not trigger eiF2α phosphorylation in HEK293T, HEp-2 and Vero E6 cells at 24 hpi (S4A Fig). Beyond that, immunofluorescence of HEp-2 cells infected for 24h showed no induction of SGs, and even some inhibition of their assembly under slight arsenite treatment (S4B Fig). These results confirm preceding data for RSV infection and suggest a new role for M2-2 as a stress granule antagonist.

### M2-2 negatively modulates cellular translation

Regardless of our prior aim in evaluating the phosphorylation of eiF2α, we observed a surprising enhancement in the expression of M2-2 under ISRIB treatment compared to non-treated cells (Fig 5C). Indeed, our initial data showed lower expression levels for both tagged M2-2 proteins when compared to FLAG-EGFP (Fig 1A), however, the mechanism of action described for ISRIB does not include stimulus of basal translation [33, 40]. To ascertain if the effect of ISRIB on M2-2 expression was specific, HEK293T cells were transfected with FLAG and EGFP-M2-2 or the control proteins FLAG-M2-1 or FLAG-YB-1, and differences between non-treated and ISRIB-treated cells were assessed by western blot. As expected, ISRIB showed no improvement in the expression of the control proteins over time, contrasting to its effect on M2-2 (S5A and S5B Fig).

**Fig 6:**
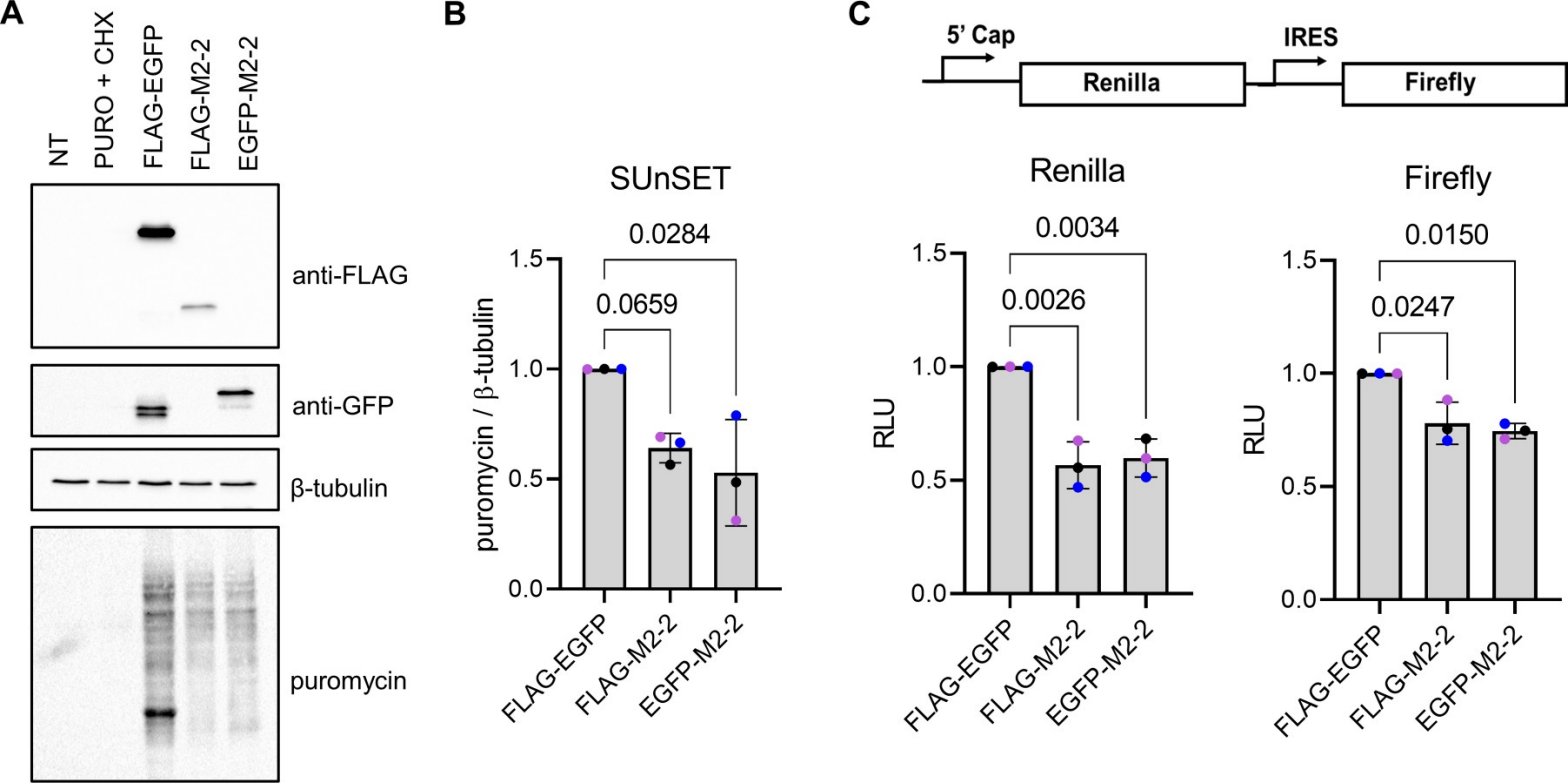
M2-2 expression downregulates translation. (A) SUnSET assay performed in HEK293T cells expressing the control FLAG-EGFP, or FLAG and EGFP-M2-2 proteins. After 24h, cells were incubated with puromycin (10 μg/mL) for 10 min, rinsed and lysed for western blot analysis. Puromycin together with cycloheximide (100 μg/mL) or non-treated cells were used as controls. Mean translation rates, indicated by puromycin staining, are quantified in (B), and all images are representative of three independent experiments (n = 3). (C) Reporter luciferase assay evaluating the inhibitory effect of M2-2 in the translation of Renilla (5’ cap) and Firefly (IRES) luciferases, as shown above. Graphs show means of the relative luminescence units (RLU) obtained in three independent experiments performed in triplicate (n = 3). Colored dots shown in (B) and (C) indicate individual means obtained in each individual experiment. Statistical analysis was performed by one-way anova followed by Dunnett’s multiple comparisons test. Error bars indicate standard deviation and p-values are indicated on the graph.

Because ISRIB prevents translation inhibition on its initial steps, we decided to examine the effect of M2-2 expression on protein syntheses, which could explain the observed effect of ISRIB on its own translation levels. Thus, FLAG-EGFP, or tagged M2-2 proteins were expressed in HEK293T cells and 24 hpt we performed a SUnSET assay to assess the translation rate measured by puromycin incorporation [24]. Curiously, both M2-2 transfected cells presented diminished translation when compared to FLAG-EGFP (Fig 6A and 6B). Additionally, we also co-expressed tagged M2-2 and FLAG-EGFP proteins with a dual luciferase reporter system containing a bicistronic 5’ cap-dependent Renilla luciferase in tandem with an IRES-dependent Firefly luciferase [23]. Supporting SUnSET results, M2-2 could hamper the expression of both luciferases, with a more prominent effect on cap-dependent translation (Fig 6C).

Unconventional mechanisms of translation are described for many viruses, including other members in the *Mononegavirales* order [41, 42]. Because RSV mRNAs are also 5’ capped, we asked if infection could interfere with the expression of the reporter luciferases. To find out if the virus can drive these inhibitory effects, we infected HEK293T cells with RSV, and 1 hpi (hour post-infection) cells were transfected with the plasmid for expression of the luciferases. Alternatively, cells were transfected before infection. Graphs in the Supplementary S6A and S6B Fig show reduced luciferase activity for both promoters in infected cells, similarly to M2-2, suggesting that RSV mRNAs might be translated through alternative paths.

### M2-2 is targeted for proteasome degradation and is directed to cytoplasmic granules during MG132 treatment

In the above results, we described two unpredicted roles for M2-2, negatively modulating translation and the cellular response to stress through eiF2α phosphorylation. Nevertheless, these functions do not directly correlate to proteomic data obtained, nor were we able to identify the granular structures to which M2-2 is recruited. Because translation elongation appears as an enriched GO term associated to M2-2 interactors (Fig 3C), we decided to investigate functional paths involved with these proteins. M2-2 potential partners encompass numerous ribosomal and chaperone proteins, in addition to the proteasome subunit PSMD11, the protein RACK1, known as a translation regulator targeted by some viruses [43, 44], and the protein VCP, recently described as necessary for proper RSV infection [45]. Among their functions, these proteins are known to participate in events related to translation elongation, ribosome quality control (RQC) and the addressing of peptides for proteasomal degradation [46–49].

**Fig 7:**
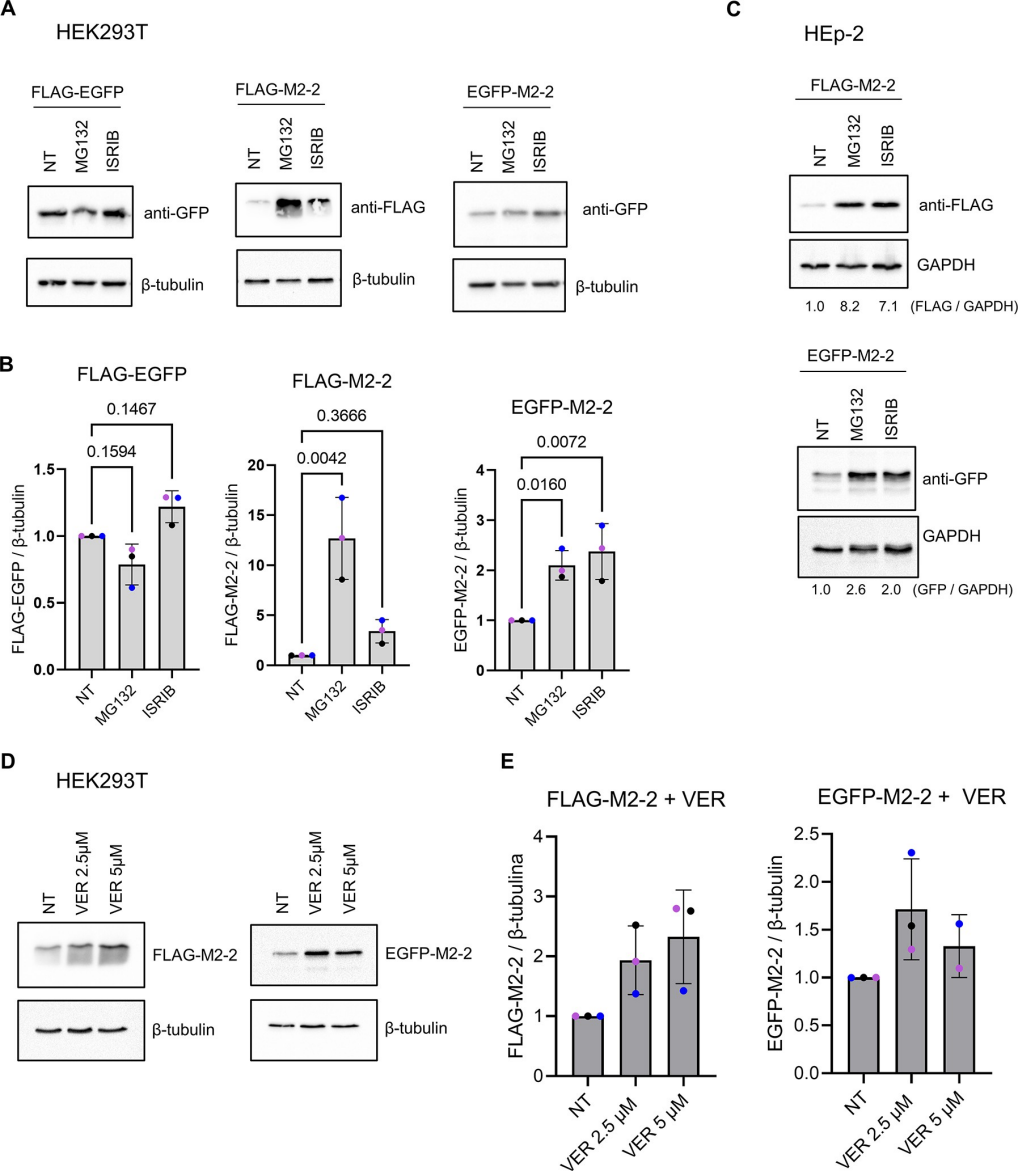
Proteasome and HSP70 inhibition promote enhanced detection of M2-2 by western blot. (A) Western blot detection of FLAG-EGFP, FLAG-M2-2 and EGFP-M2-2 expressed for 24h in HEK293T cells non-treated (NT), or under ISRIB (200 nM) and MG132 (5 μM) treatments. Images are representative of three independent experiments. Graphs in (B) present the mean values quantified in (A). Statistical analysis was performed by one-way anova followed by Dunnett’s multiple comparisons test and p-values are indicated on the graph. (C) Expression of FLAG and EGFP-M2-2 in HEp-2 cells, as described for HEK293T cells. Normalized expression of FLAG and EGFP-M2-2 is shown below. (D) Expression of FLAG and EGFP-M2-2 under VER treatment (NT, 2.5 or 5 μM). Western blots are quantified in (E) and are representative of three independent experiments. In the graphs, colored dots indicate values from independent replicates (n = 3), and error bars show standard deviation.

**Fig 8:**
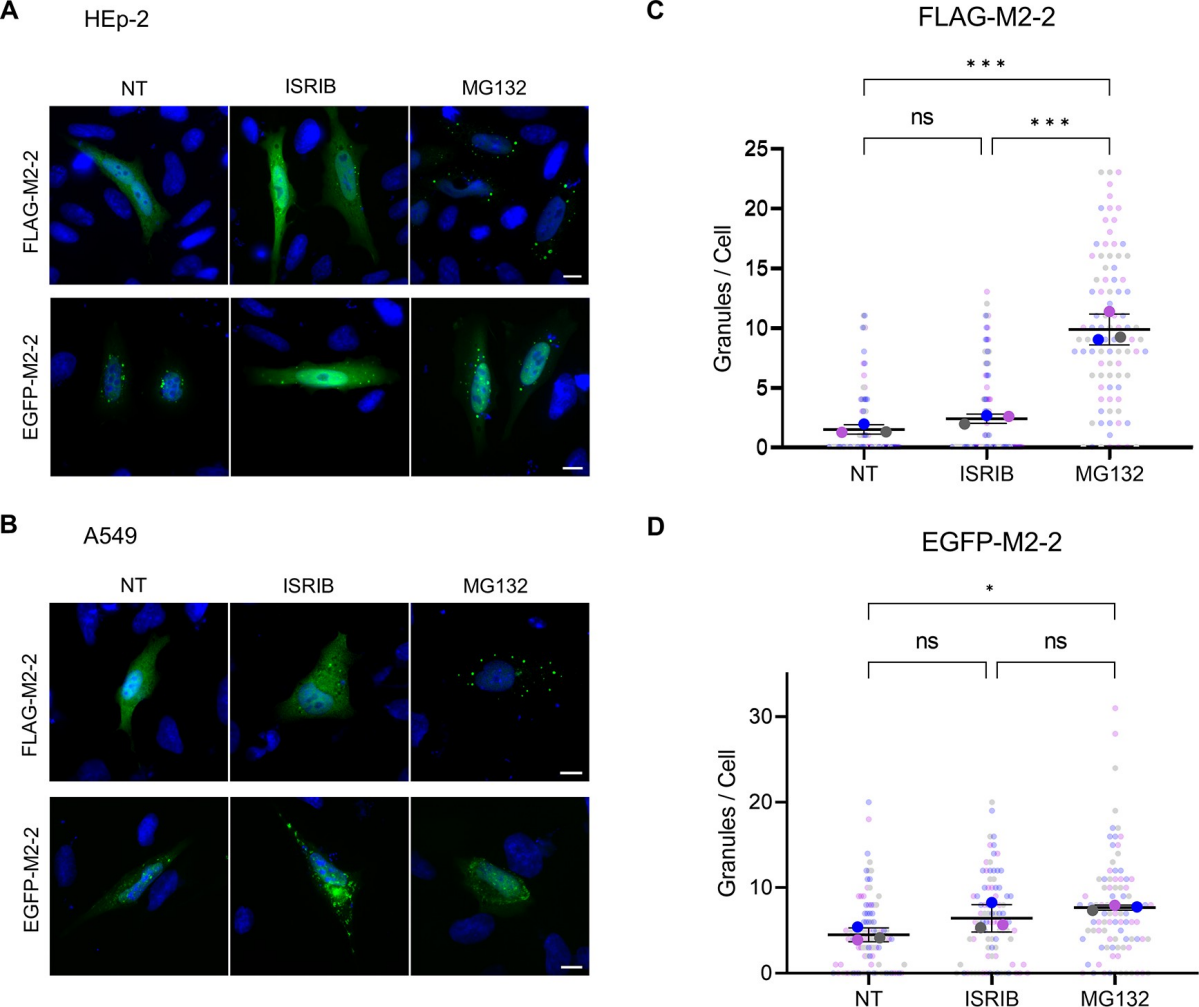
MG132 treatment induces the recruitment of FLAG-M2-2 into cytoplasmic granules. Cellular distribution of FLAG-M2-2 and EGFP-M2-2 under different treatments (ISRIB 200 nM for 20h, MG132 5 μM for 2h) in HEp-2 (A) or A549 cells (B), showing increase in the number of granules stained by FLAG-M2-2 under MG132 treatment. Graphs in (C) and (D) present the quantification in HEp-2 cells of the average number of granules for FLAG and EGFP-M2-2 during each treatment. Small dots show the individual number of counted granules over 30 cells for each condition, while bigger dots present mean values. Different colors show values obtained across each independent experiment (n = 3). Statistical differences were evaluated by one-way anova following Tukey’s multiple comparisons test. Error bars indicate standard deviation, and p-values are indicated by * p < 0.05 and *** p < 0.001, ns = not significant.

Considering these observations and the reduced protein mass detected for M2-2 by western blot, we decided to investigate the involvement of the protein with the proteasomal machinery. For that, HEK293T cells were transfected with FLAG-EGFP or tagged M2-2 proteins following treatment with the proteasome inhibitor MG132, using as controls ISRIB or non-treated cells. While no difference was observed in the expression of FLAG-EGFP between treatments, MG132 improved the detection of both FLAG and EGFP-M2-2, with a more distinguishable effect in FLAG-M2-2 expression (Fig 7A and 7B). Similar results were obtained in HEp-2 cells treated with MG132 or ISRIB (Fig 7C), or during the treatment of HEK293T cells with the HSP70 inhibitor VER, showing that the activity of this chaperone also contributes for the degradation of M2-2 (Fig 7D and 7E). In addition to western blot data, we also verified that MG132, but not ISRIB, is able to induce the recruitment of FLAG-M2-2 into cytoplasmic granules in either HEp-2 or A549 cells, whereas only a modest effect was observed for EGFP-M2-2 (Fig 8A–8D).

### M2-2 colocalizes with granules composed of defective ribosomal products

**Fig 9:**
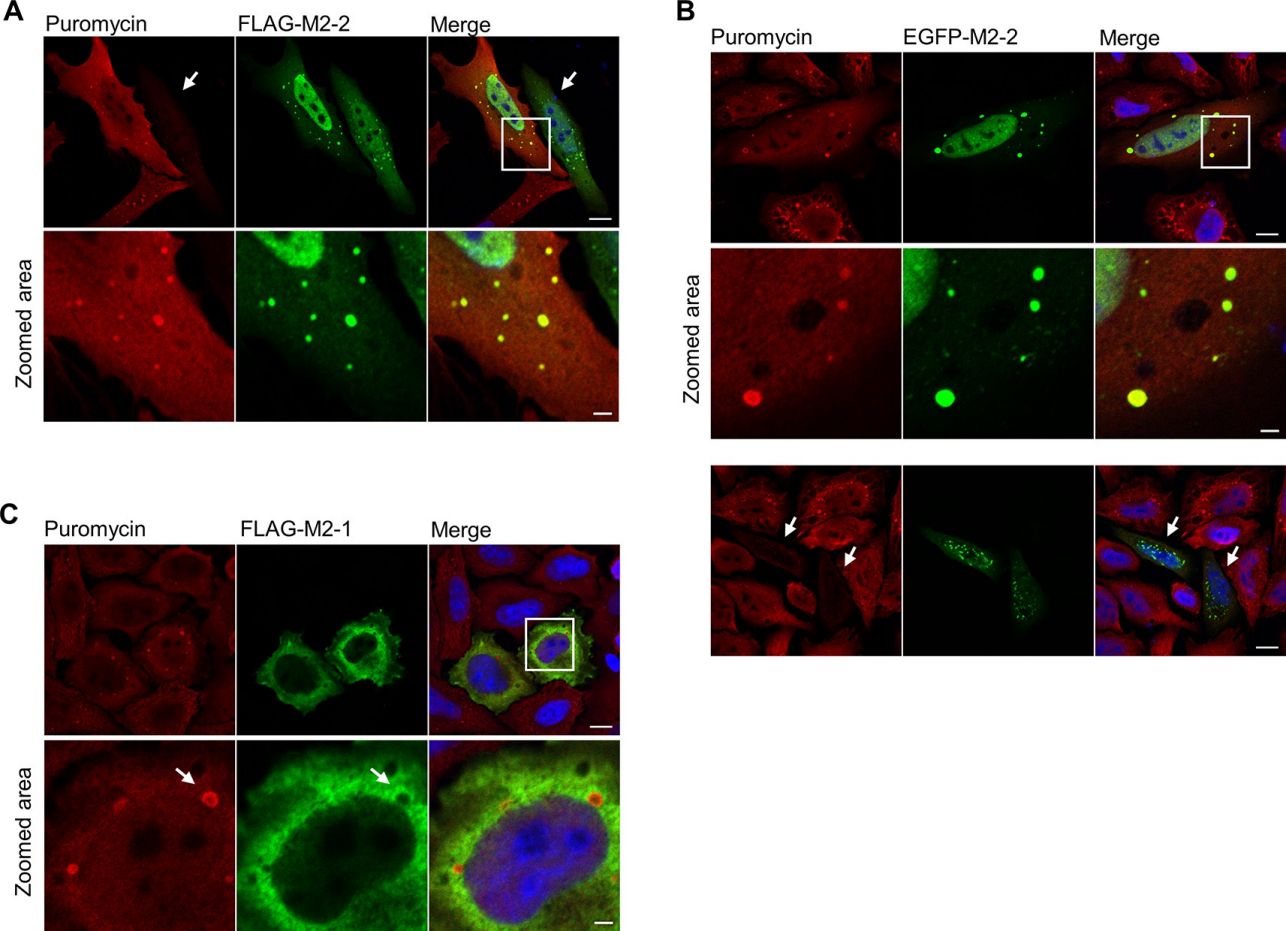
M2-2 interacts with defective proteins targeted for proteasome degradation. FLAG-M2-2 (A) and EGFP-M2-2 (B) were expressed in HEp-2 cells for 24h, and then cells were incubated with puromycin (10 μg/mL) and MG132 (5 μM) for 2h. After treatment, cells were rinsed, fixed, and stained with anti-puromycin for tracking of DRiPs, showing colocalization with M2-2. Inhibition of translation can be observed in cells presenting low puromycin incorporation (white arrows in (A) and (B)–third row). (C) As a control, FLAG-M2-1 was expressed and submitted to the same treatments. White arrows in the zoomed area show absence of colocalization between puromycin stained granules and FLAG-M2-1. All images were taken with a Zeiss LSM-780-NLO microscope and are representative of three independent experiments. Scales bars are 10 μm for full size panels, and 2 μm for zoomed panels.

Previous reports in the literature have shown that proteasome inhibition can trigger aggregation of ubiquitinated peptides addressed for proteasome-dependent degradation, which can be tracked by puromycin-induced termination of translation [50, 51]. To examine if these are the same structures to which M2-2 is recruited, HEp-2 cells were transfected with FLAG-M2-2 or EGFP-M2-2, and 24 hours post-transfection cells were treated with puromycin and MG132 for 2h. As a negative control, cells were transfected with FLAG-M2-1 following the same treatments. Fig 9 shows colocalization between puromycin granules and both M2-2 tagged proteins, contrasting to FLAG-M2-1, for which colocalization was absent (Fig 9A–9C). Furthermore, we could detect cells where puromycin incorporation was inhibited (Fig 9A and 9B–white arrows), confirming our preceding results for inhibition of translation. Similar results were also obtained in Vero E6 cells, with FLAG and EGFP-M2-2 exhibiting colocalization with puromycin granules (S7A Fig), as well as inhibition of protein synthesis evidenced by low puromycin incorporation (S7B Fig).

### M2-2 localizes with light ribosomal fractions and endoplasmic reticulum

**Fig 10:**
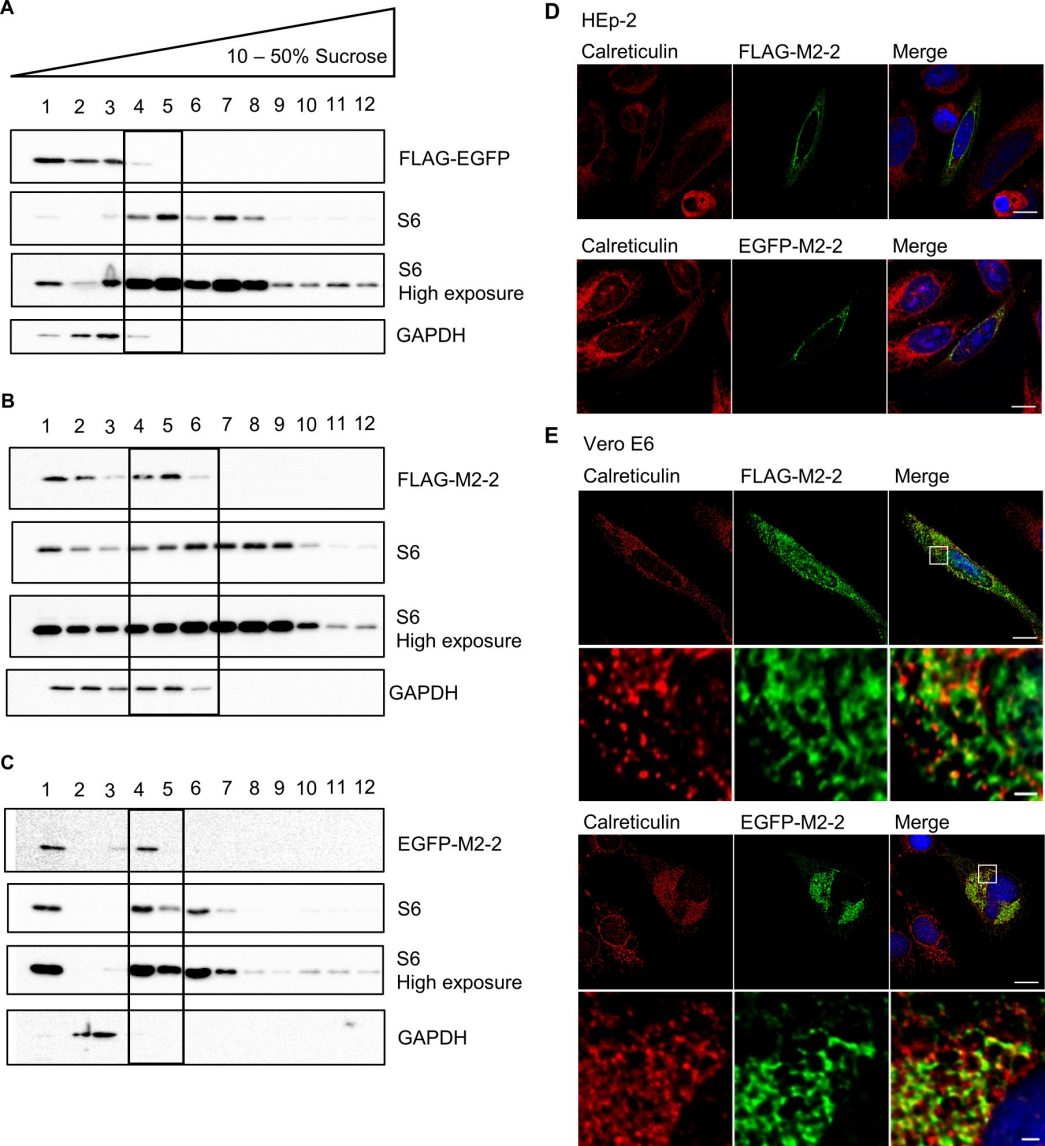
M2-2 shows proximity with the ribosomal machinery. (A) HEK293T cells expressing FLAG-EGFP, FLAG-M2-2 (B) or EGFP-M2-2 (C) for 24h were submitted to cell fractionation through sucrose gradient. Staining with the proteins GAPDH and S6 were used to determine soluble and ribosomal fractions. Images show that FLAG and EGFP-M2-2 can be found in light ribosomal fractions, as highlighted in the blots (fractions 4–6). Otherwise, FLAG-EGFP were detected only in soluble fractions (1–3). (D) FLAG-M2-2 and EGFP-M2-2 were expressed in HEp-2 or Vero E6 cells (E) and analyzed by confocal immunofluorescence against the ER marker calreticulin, showing colocalization between the proteins and this structure. Images are representative of three independent experiments. Zoomed areas in (E) show the proximity between M2-2 and calreticulin in higher detail. Scale bars are 10 μm for full size panels and 1 μm for zoomed areas.

The ability of M2-2 to inhibit translation and its recruitment to DRiPs, followed by proteasome degradation, raise questions about whether these observations could result from the interaction of M2-2 with the ribosomal machinery. To explore this possibility, we performed sucrose fractionation of HEK293T cells expressing FLAG and EGFP-M2-2, or the control protein FLAG-EGFP, using as a marker for ribosomal fractions the protein S6 (Fig 10A–10C). Curiously, tagged M2-2 proteins were detected in both soluble and lighter ribosomal fractions, while FLAG-EGFP remained in the soluble phase.

Besides being diffuse in the cytoplasm, ribosomes can also be found associated to the endoplasmic reticulum (ER) membrane. Indeed, we have observed that in some cells, the perinuclear distribution of M2-2 resembled that of the ER (Fig 1B, S1 Fig). To verify if M2-2 were also recruited to this site, we performed confocal analysis of HEp-2 and Vero E6 transfected cells using as an ER marker the protein calreticulin. Fig 10D and 10E show that either FLAG or EGFP-M2-2 colocalized with ER, reinforcing the proximity of M2-2 with the ribosomal machinery.

### M2-2 modulates translation without triggering proteostatic stress

Lastly, we evaluated whether the involvement of M2-2 with ribosomal and proteasomal machineries, as well as its inhibitory effect on translation, could modulate the expression of other related proteins. To do so, we expressed FLAG-EGFP or M2-2 proteins in HEK293T cells under no treatment, or in the presence of ISRIB and MG132. Quantitative analysis showed no difference in the expression of the chaperones HSP70 and BiP (S8A–S8C Fig), indicating that the inhibitory effect of M2-2 does not trigger the unfolded protein response [50]. Furthermore, we noticed that M2-2 expression increased the amount of the phosphorylated 4E-BP1 protein (S8A and S8D Fig), which contrasts with the observed inhibition of translation [52]. Finally, the comparison of ubiquitin levels between ISRIB and non-treated cells also suggests it does not affect proteasome activity, reinforcing that MG132 and ISRIB regulate M2-2 expression by different mechanisms (S9A and S9B Fig). Together, these results show that though M2-2 does not induce cellular responses to unfolded peptides, it can modulate proteins involved with translation initiation, as observed for 4E-BP1 and eiF2α.

## Discussion

In this work, we investigated the yet unexplored relationship between the RSV M2-2 protein and the cellular machinery. In our initial experiments, the interactome of M2-2 showed a substantial correlation with the regulation of translation and protein folding. This was later confirmed by the ability of M2-2 to inhibit translation, as well as the integrated stress response, mediated by the phosphorylation of eiF2α and SGs assembly. Furthermore, we also verified that M2-2 is targeted for proteasome degradation, and colocalizes with defective ribosomal products, suggesting it can be involved with translation elongation as well. To our knowledge, this is the first study to describe additional functions performed by M2-2 to modulate the cellular metabolism, proposing new roles for this protein during RSV infection.

In our proteomic analysis, we identified 72 potential cellular partners of M2-2, composed by three main functional clusters, related to mRNA splicing, translation and protein folding (Fig 3B, S1 Table). This is in accordance with our observations exploring the cellular distribution of M2-2 (Fig 1B, S1 Fig). In the nucleus, we detected M2-2 diffusely in the nucleoplasm or in a punctate pattern, resembling what is observed for some splicing effectors [53, 54]. Other RSV proteins have been described to localize into the nucleus acting as inhibitors of transcription [17, 18], with no data exploring their direct relation with mRNA splicing. However, if M2-2 is recruited to sites where splicing takes place, and if it interferes with pre-mRNA processing, remains to be addressed.

In the cytoplasm, M2-2 was only partially recruited to viral inclusion bodies in infected cells, presenting mainly diffuse and granular distributions (Fig 2A and 2B). The recruitment of M2-2 to granules distinct from IBs was similar to previous reports exploring the relationship between these structures and stress granules [37–39]. Because some of the M2-2 partners are also described as SG components [32], we first sought to investigate the relationship of the protein with SGs, among other cellular granules (Fig 4, S2 Fig). Though no colocalization has been observed for M2-2 granules and the SG marker G3BP1, we could characterize the ability of M2-2 in inhibiting the assembly of stress granules in different cell lines (Fig 5A and 5B, S3A–S3D Fig). Accordingly, we also found reduced levels of phosphorylated eiF2α in M2-2 expressing cells (Fig 5C and 5D).

Likewise, we did not detect triggering of eiF2α phosphorylation or SGs assembly in RSV infected cells after 24h (S4A and S4B Fig). In fact, it has been previously shown that RSV infection prevents the activation of the integrated stress response by impairing the activity of the eiF2α kinase PKR, which depends on the viral nucleoprotein [37]. These observations are in agreement with the results obtained for M2-2, suggesting it may contribute to antagonize host defenses coordinated by eiF2α during infection. However, further experiments are necessary to determine the functions performed by the protein in this context.

Moreover, we were also surprised by the improvement in the expression of M2-2 under ISRIB treatment. ISRIB acts on the eiF2B –eiF2 ternary complex– 43S preinitiation complex branch [33, 40, 52], preventing the inhibitory effect of the phosphorylated eiF2 α-subunit on the eiF2B guanine exchange factor, restoring basal levels of translation, but not stimulating overexpression of proteins [33]. Indeed, we confirmed the specificity of ISRIB for M2-2 in comparison to the absence of effect in the expression of control proteins (S5A and S5B Fig), indicating that M2-2 could interfere with this path, inhibiting its own translation as a consequence. However, except for the identification of RACK1 (also identified as GNB2L1), described as a regulator of translation initiation [43, 44], the other proteins identified by proteomic analysis (Fig 2B, S1 Table), though related to translation, are not described as main effectors during initiation of translation, nor part of the path regulated by ISRIB [52]. Besides, analysis of enriched GO terms pointed translation and translation elongation, but not translation initiation, as functions performed by M2-2 potential partners (Fig 2C). Even if these findings are not totally in agreement, our results revealed a new role for M2-2 as an inhibitor of translation. This was verified by SUnSET assay, showing reduced translation rates measured by puromycin incorporation, as well as during the co-expression of M2-2 with a luciferase reporter system, showing that it was able to inhibit both 5’-cap and IRES-dependent translations (Fig 6A–6C).

Since RSV transcripts are also 5’ capped, we sought to investigate whether infection could lead to reduced translation of both luciferase reporters, obtaining similar results to those of M2-2 (S6A and S6B Fig). In the course of infection, many viruses take control of the cellular machinery to facilitate the expression of their own proteins. Since RSV transcripts are flanked by different 5’ UTR sequences, one possibility is that the virus can favor the production of its proteins through alternative translation pathways. Such mechanisms are described for some cellular proteins and different viruses, including poliovirus and hepatitis C virus, as well as RSV related virus such as VSV, rabies virus and measles virus [41, 42, 55, 56]. Besides, the nuclear transcription of M2-2 under a CMV promoter generates mRNAs containing ordinary 5’ UTRs rather than viral regulatory sequences, which could lead to its downregulated expression. However, it remains to be elucidated whether the expression of M2-2 flanked by RSV 5’ UTR sequences presents normal expression levels.

Finally, looking for a direct correlation between proteomic data and the distribution of M2-2 into granules, we verified that the protein is targeted for proteasome degradation in human cells, which can be inhibited by treatments with MG132 or the HSP70 inhibitor VER (Fig 7A–7E). Beyond that, we could detect improved recruitment of FLAG-M2-2 to granules when proteasome activity was inhibited, suggesting that these structures were cleared by this degradation path (Fig 8A–8D). It is worth noting that compared to FLAG-M2-2, EGFP-tagged M2-2 exhibits a more granular pattern in the cytoplasm (Fig 8C and 8D), indicating its granules are not properly cleared. In agreement with this observation, EGFP-M2-2 expression showed a modest effect under MG132 treatment in comparison to FLAG-M2-2 (Fig 7A and 7B). Seeing that M2-2 is a small 10 kDa protein, fusing it to EGFP could interfere with some of its interactions, therefore preventing its proper association with proteasome effectors.

In the course of peptide elongation, stalled ribosomes or incorrect folding of newly synthesized proteins can signal for ribosome quality control (RQC) effectors to ubiquitinate and dismount 80S ribosomal subunit, addressing nascent peptides for proteasome degradation, a process assisted by accessory chaperones [57–59]. Furthermore, literature reports describe the accumulation of cytoplasmic granules composed of defective ribosomal products (DRiPs) when proteasome activity is inhibited [50, 51]. As M2-2 was shown to depend on both proteasome and HSP70 activities to be degraded, we asked if the protein was addressed to DRiPs in the cytoplasm. This was confirmed by immunofluorescence (Fig 9, S7 Fig), showing the colocalization of both FLAG and EGFP-M2-2 with these granules. Additionally, the interaction of M2-2 with DRiPs, as well as its association with the proteasome machinery, implied it could be involved with translation elongation, directing prematurely terminated peptides for degradation, in a similar way to viruses that utilize RQC induction to favor translation of its own transcripts [46, 47, 60]. Consistent with this possibility, we verified the presence of M2-2 in light ribosomal fractions after sucrose fractionation and the colocalization of the protein with endoplasmic reticulum, a site of active translation (Fig 10).

In line with these results, RQC has been described as an eiF2α antagonist [61], and the premature termination of elongation could contribute for the observed decrease in translation measured in both SUnSET and reporter luciferase assays. Besides, we did not detect induction of the unfolded protein response in M2-2 expressing cells, but we did observe increased levels of phosphorylated 4E-BP1 (S8A–S8D Fig). It raises the question whether the modulation in the phosphorylation of eiF2α and 4E-BP1 are cellular responses to translation inhibition and induction of RQC rather than direct consequences of M2-2 expression. Moreover, it has been shown that the knockdown of G3BP1 impairs RSV replication, even if infection prevents SGs assembly [39]. This effect could be explained by the reduction in the recycling of ubiquitinated ribosomes after RQC induction, since G3BP1 also acts in this path [62]. Regardless of these possibilities, whether M2-2 expression or RSV infection can actively promote RQC or promote premature termination of translation, remains to be addressed.

We point out that the expression of tagged proteins in the absence of infection is a limitation of this study. Therefore, further experiments are necessary to validate our data for the M2-2 protein expressed by the virus. Despite the limitations imposed by the lack of antibodies against M2-2, our findings are strengthened by the consistent results obtained using two different tagged proteins over different cell lines. This allowed us to explore additional functions performed by the RSV M2-2 protein for the first time. Our observations indicate that M2-2 is involved in different events associated with protein synthesis, modulating regulators of its initiation, inhibiting translation and interacting with peptides targeted for degradation. These findings suggest new mechanisms employed by the virus to subvert the cell machinery to its own favor.

## Supporting information

S1 Fig Cellular distribution of FLAG and EGFP-M2-2 in Vero E6 cells. Immunofluorescence of FLAG-M2-2 and EGFP-M2-2 expressed for 24h in Vero E6 cells, presenting distribution patterns similar to that observed in HEp-2 cells, as indicated above (Fig 1B). Images are representative of three independent experiments. Scale bars 10 μm.
(TIF)

S2 Fig M2-2 does not colocalize with tagged YB-1 proteins, marker of SGs and p-bodies. (A) Confocal immunofluorescence performed in HEp-2 cells showing the co-expression of FLAG-M2-2 and EGFP-YB-1, a protein recruited for both SGs and p-bodies. Zoomed area presents raw and de-convolved images, where M2-2 granules and p-bodies (stained by EGFP-YB-1) can be distinguished as different structures. (B) Confocal immunofluorescence of cells co-expressing FLAG-YB-1 and EGFP-M2-2, as described in (A). Images are representative of three independent experiments. Scale bars are 10 μm for full size panels and 2 μm for zoomed areas.
(TIF)

S3 Fig M2-2 prevents the assembly of SGs in different cell lines. HEp-2 cells expressing FLAG-M2-2 (A) and EGFP-M2-2 (B) were treated or not with arsenite (0.5 mM for 30 min) and stained against the protein PABP, showing the inhibition of SGs assembly for a different SG marker. Alternatively, inhibition of SGs, stained by G3BP1, is also shown in A549 (C) and Vero E6 cells (D), which is reproduced for both FLAG-M2-2 and EGFP-M2-2. Both proteins were expressed for 24h. Arsenite-treated cells (A549–0.5 mM for 30 min, Vero E6–1 mM for 1h) are indicated on the left. All images are representative of three independent experiments. Scale bars 10 μm.
(TIF)

S4 Fig RSV infection does not trigger eiF2α phosphorylation or SGs assembly. (A) Western blot detection of phospho-eiF2α in HEK293T, HEp-2 and Vero E6 cells mock or RSV infected for 24h. Phos-pho-eiF2α / total eiF2α ratios are shown below. Infection is indicated by detection of the RSV P protein. (B) HEp-2 cells were mock, or RSV infected for 24h and submitted or not to arsenite treatment for 30 min at the indicated concentrations (NT, 0.25 mM or 0.5 mM). SGs were stained by G3BP1 while RSV infected cells were detected with anti-N. Impairment of SGs assembly in infected cells can be seen in all panels. Images were taken with a widefield ZEISS Axio Vert.A1 microscope and are representative of three independent experiments. Scale bars 10 μm.
(TIF)

S5 Fig ISRIB treatment enhances M2-2 expression. (A) Time-course expression of FLAG-M2-2 and EGFP-M2-2 in HEK293T performed in duplicate. Cells were ISRIB treated (200 nM) or not (NT–non-treated) and collected at indicated times for western blot analysis. As controls, cells were transfected with FLAG-M2-1 or FLAG-YB-1. Quantification of the detected bands is shown in graphs on (B), showing the specific effect of ISRIB in the expression of M2-2.
(TIF)

S6 Fig RSV infection inhibits both 5’ cap and IRES-dependent translations. (A) HEK293T cells were mock or RSV infected, and 1 hpi cells were transfected with the vector for expression of reporter luciferases (indicated above). 24 hpi, cells were lysed, and luminescence activity was evaluated, showing inhibition of both Renilla (5’ cap) and Firefly (IRES) luciferases. (B) Alternatively, inhibition was also seen in cells transfected for 4h before infection, following lysis at 20 hpi (as indicated above). Colored dots in the graphs indicate individual means of two independent experiments performed in triplicate (n = 2). Error bars show standard deviation.
(TIF)

S7 Fig M2-2 colocalizes with DRiPs and inhibits translation in Vero E6 cells. (A) FLAG-M2-2, EGFP-M2-2 and FLAG-M2-1 were expressed in Vero E6 cells and treated with puromycin (10 μg/mL) and MG132 (5 μM) for 2h. Both FLAG and EGFP-M2-2 colocalized with puromycin granules in these cells, with no recruitment of FLAG-M2-1 to puromycin granules. (B) Images show cells were M2-2 expression could inhibit puromycin incorporation, indicating that M2-2 ability to hamper translation is kept in this cell line. All images were taken with a ZEISS Axio Vert.A1 microscope and are representative of two independent experiments. Scale bars 10 μm.
(TIF)

S8 Fig M2-2 does not enhance the expression of chaperones but modulates the phosphorylation of translation effectors. (A) FLAG-EGFP or FLAG and EGFP-M2-2 proteins were expressed in HEK293T under the following treatments, NT–non-treated, MG132 (5 μM), ISRIB (200 nM or 500 nM). After 24h, cells were lysed and extracts were analyzed by western blot, looking for changes in the expression of proteostatic stress markers (HSP70 and BiP), as well as regulators of translation initiation (phospho-4E-BP1). Ubiquitin levels were also accessed for evaluation of proteasome inhibition by MG132. The normalized expression of the proteins is shown as means on the graphs for HSP70 (B), BiP (C) and phosphor-4E-BP1 (D). Statistical differences were evaluated by two-way anova followed by Dunnett’s multiple comparisons test. Colored dots in all graphs indicate in-dividual values from paired independent experiments (n = 3). Error bars indicate standard deviation and p-values are shown in the graphs.
(TIF)

S9 Fig ISRIB treatment does not induce enhanced ubiquitin detection. (A) HEK293T cells expressing FLAG-EGFP or FLAG and EGFP-M2-2 proteins were treated or not with ISRIB (as indicated above, NT—non-treated) and had their ubiquitin levels assessed by western blot. Quantitative analysis of three independent experiments is shown as means on the graph in (B), with no significant differences detected between different treatments. Statistical differences were evaluated by two-way anova followed by Dunnett’s multiple comparisons test. Colored dots in graphs indicate individual values from paired independent experiments (n = 3). Error bars indicate standard deviation and p-values are shown in the graphs.
(TIF)

S1 Table Mass spectrometry and enrichment analysis. (XLSX)

S1 File DNA sequence of optimized genes. (DOCX)

S1 Raw images (PDF)
