# Supplementary figures and images for: The respiratory syncytial virus M2-2 protein is targeted for proteasome degradation and inhibits translation and stress granules assembly
Source: PLoS One. 2023 Jul 25;18(7):e0289100. doi: 10.1371/journal.pone.0289100 (PMC10368288; doi:10.1371/journal.pone.0289100)

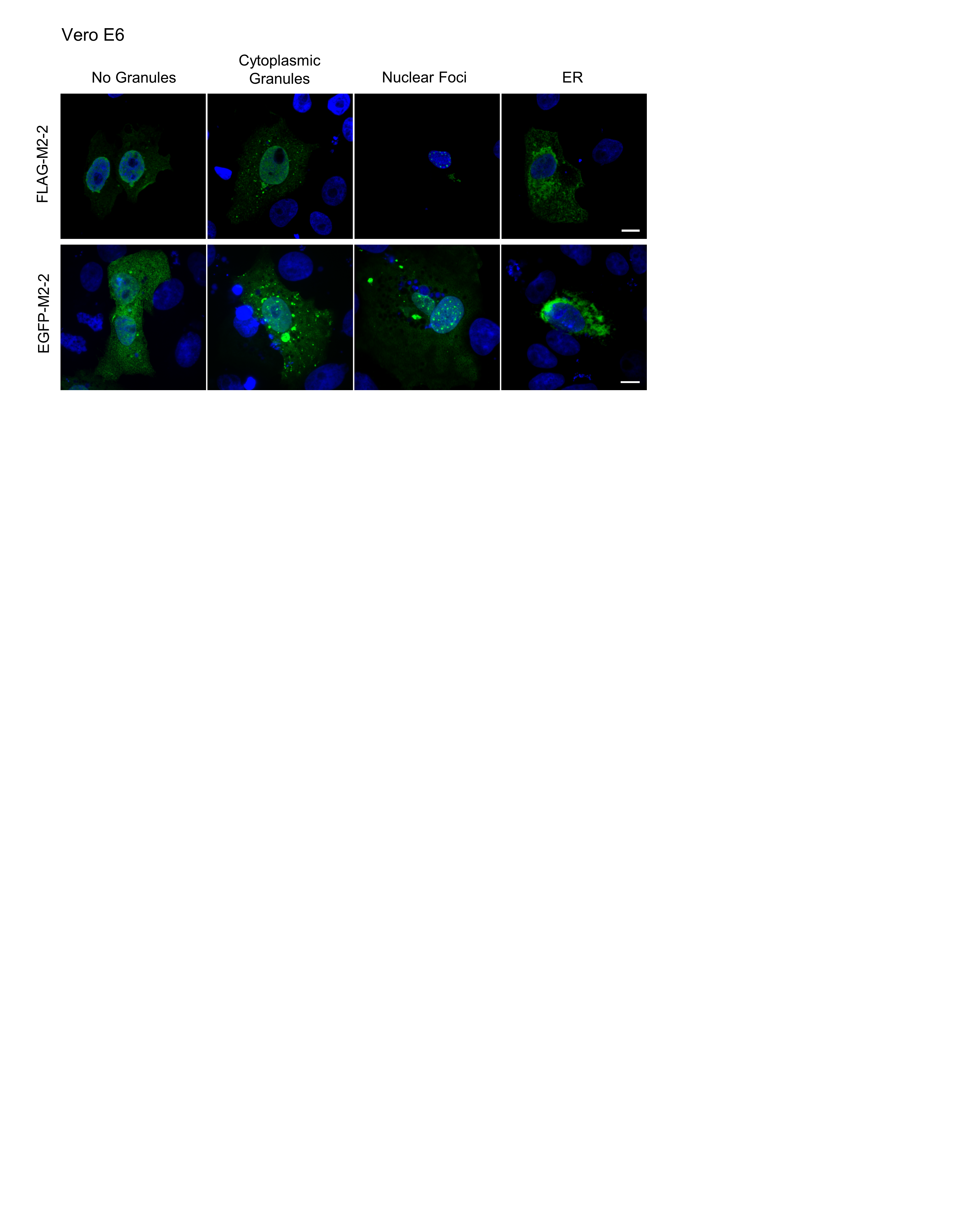

Supplement: S1 Fig — Immunofluorescence of FLAG-M2-2 and EGFP-M2-2 expressed for 24h in Vero E6 cells, presenting distribution patterns similar to that observed in HEp-2 cells, as indicated above (Fig 1B). Images are representative of three independent experiments. Scale bars 10 μm. (TIF) [file pone.0289100.s001.TIF]

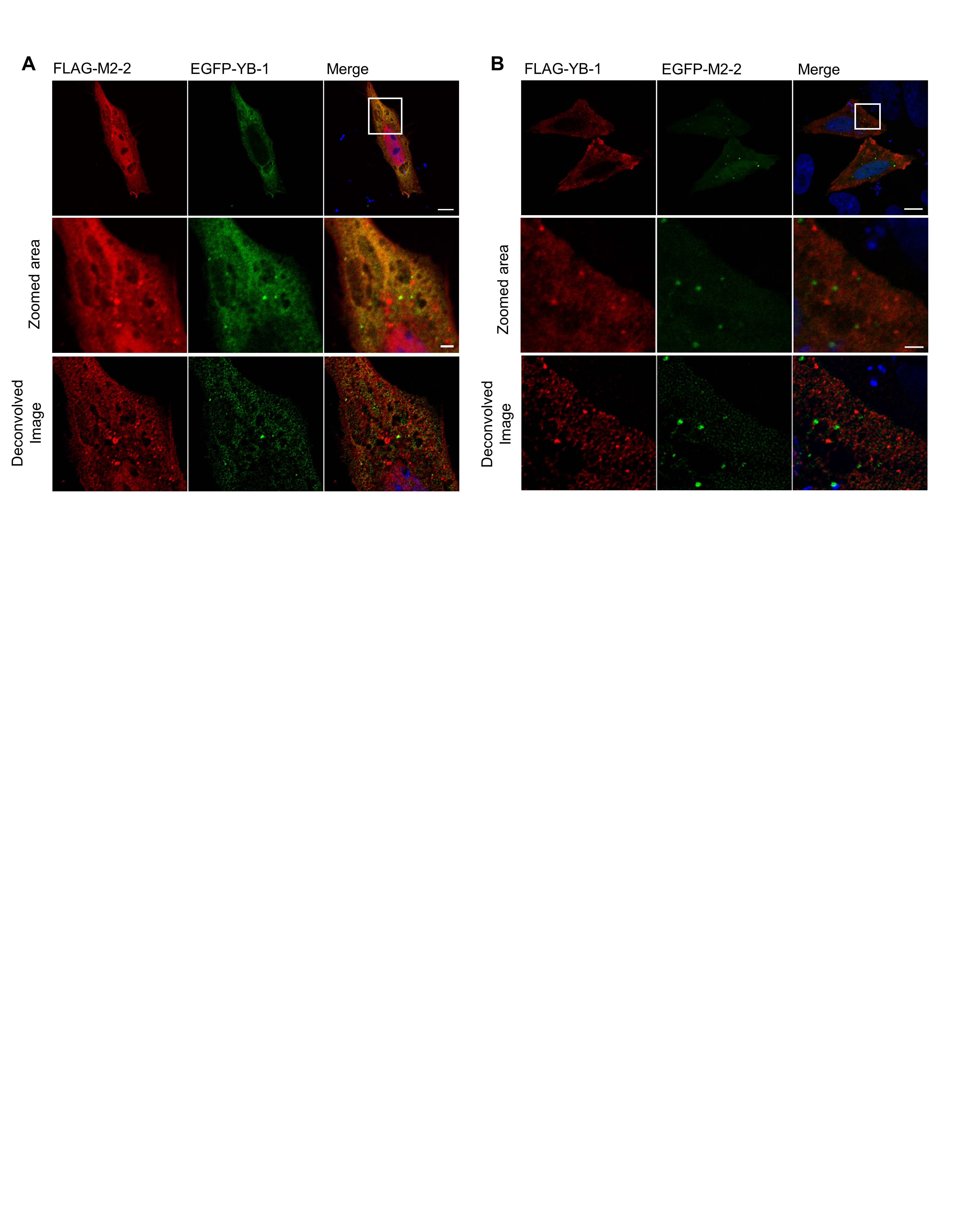

Supplement: S2 Fig — (A) Confocal immunofluorescence performed in HEp-2 cells showing the co-expression of FLAG-M2-2 and EGFP-YB-1, a protein recruited for both SGs and p-bodies. Zoomed area presents raw and de-convolved images, where M2-2 granules and p-bodies (stained by EGFP-YB-1) can be distinguished as different structures. (B) Confocal immunofluorescence of cells co-expressing FLAG-YB-1 and EGFP-M2-2, as described in (A). Images are representative of three independent experiments. Scale bars are 10 μm for full size panels and 2 μm for zoomed areas. (TIF) [file pone.0289100.s002.TIF]

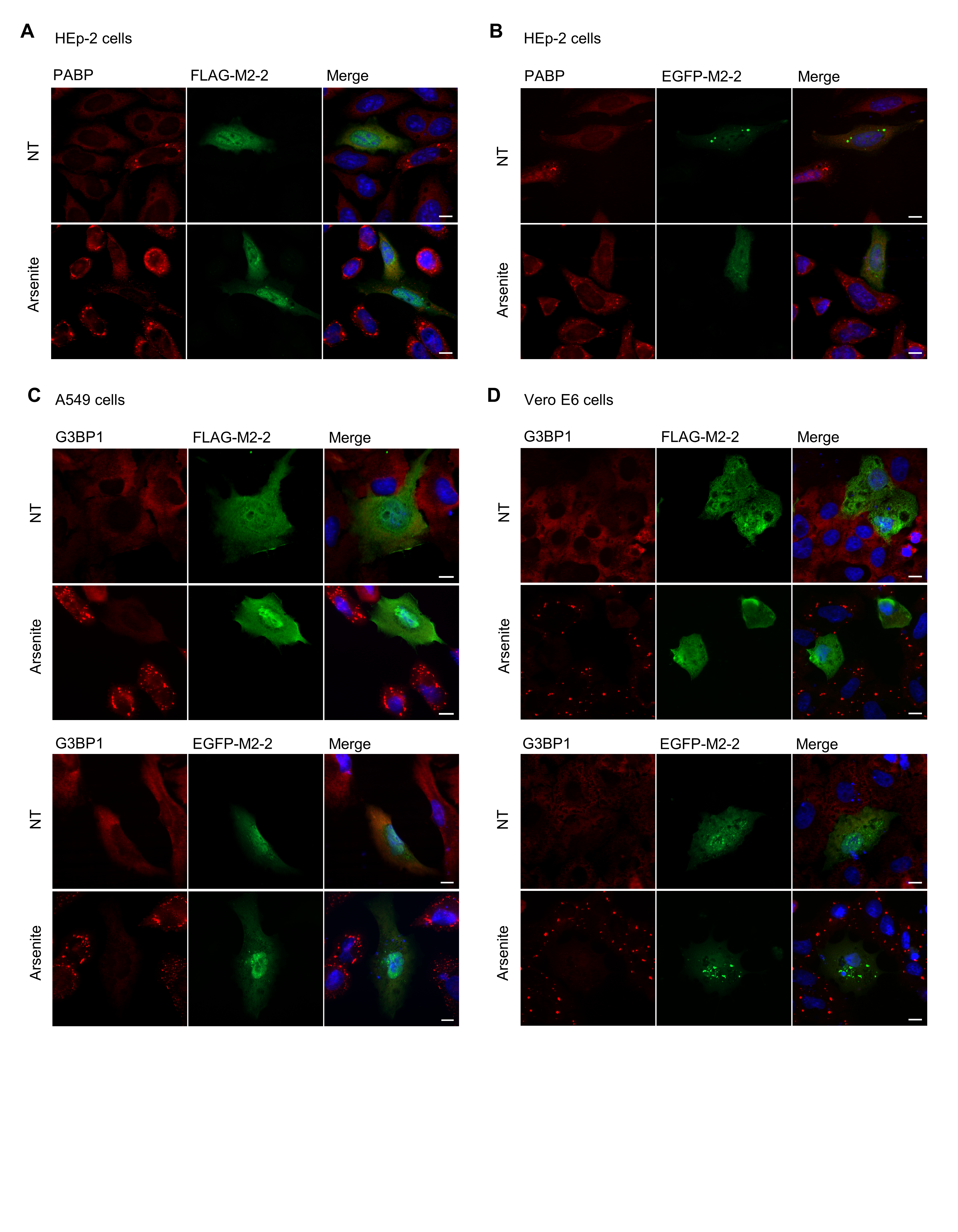

Supplement: S3 Fig — HEp-2 cells expressing FLAG-M2-2 (A) and EGFP-M2-2 (B) were treated or not with arsenite (0.5 mM for 30 min) and stained against the protein PABP, showing the inhibition of SGs assembly for a different SG marker. Alternatively, inhibition of SGs, stained by G3BP1, is also shown in A549 (C) and Vero E6 cells (D), which is reproduced for both FLAG-M2-2 and EGFP-M2-2. Both proteins were expressed for 24h. Arsenite-treated cells (A549–0.5 mM for 30 min, Vero E6–1 mM for 1h) are indicated on the left. All images are representative of three independent experiments. Scale bars 10 μm. (TIF) [file pone.0289100.s003.TIF]

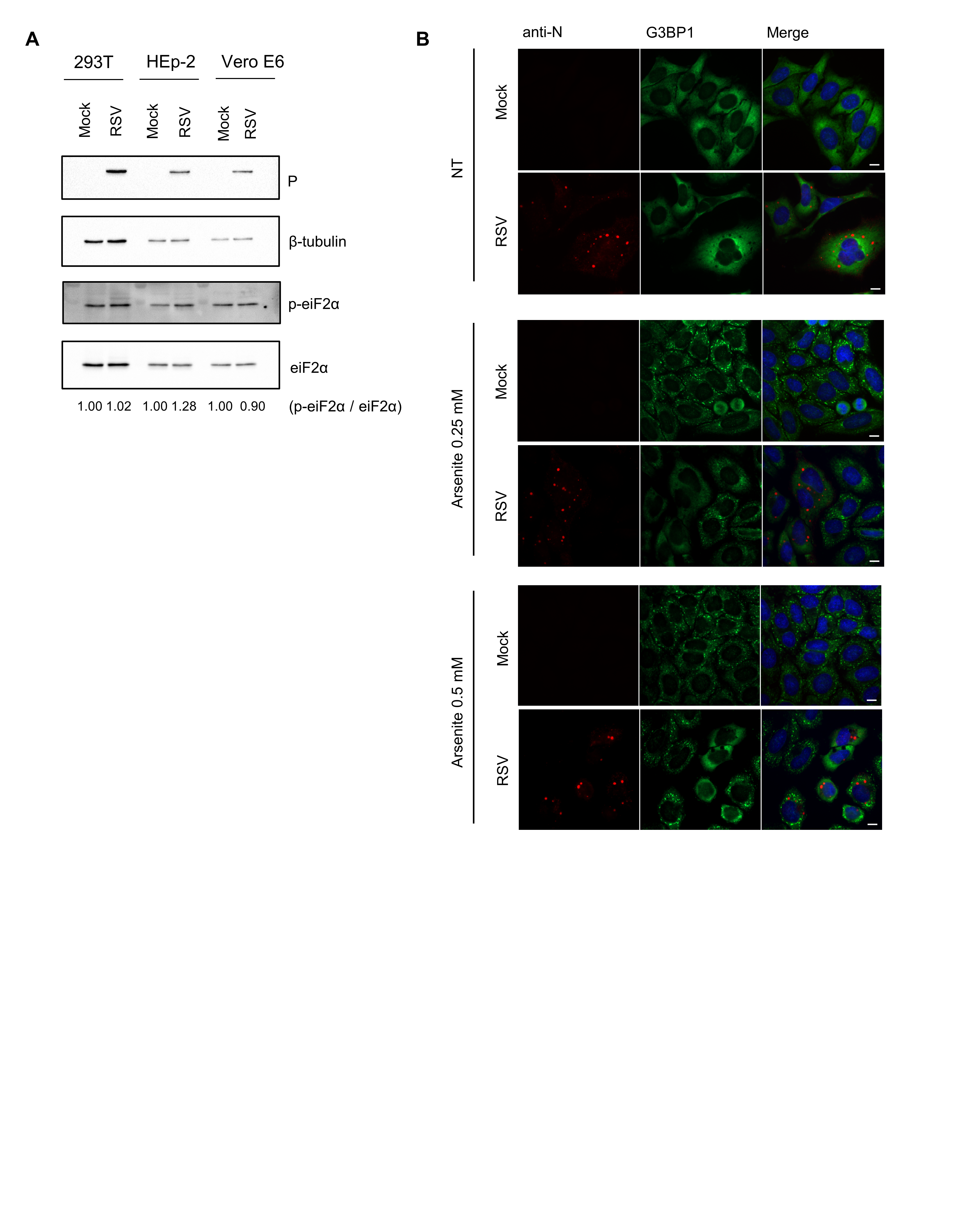

Supplement: S4 Fig — (A) Western blot detection of phospho-eiF2α in HEK293T, HEp-2 and Vero E6 cells mock or RSV infected for 24h. Phos-pho-eiF2α / total eiF2α ratios are shown below. Infection is indicated by detection of the RSV P protein. (B) HEp-2 cells were mock, or RSV infected for 24h and submitted or not to arsenite treatment for 30 min at the indicated concentrations (NT, 0.25 mM or 0.5 mM). SGs were stained by G3BP1 while RSV infected cells were detected with anti-N. Impairment of SGs assembly in infected cells can be seen in all panels. Images were taken with a widefield ZEISS Axio Vert.A1 microscope and are representative of three independent experiments. Scale bars 10 μm. (TIF) [file pone.0289100.s004.TIF]

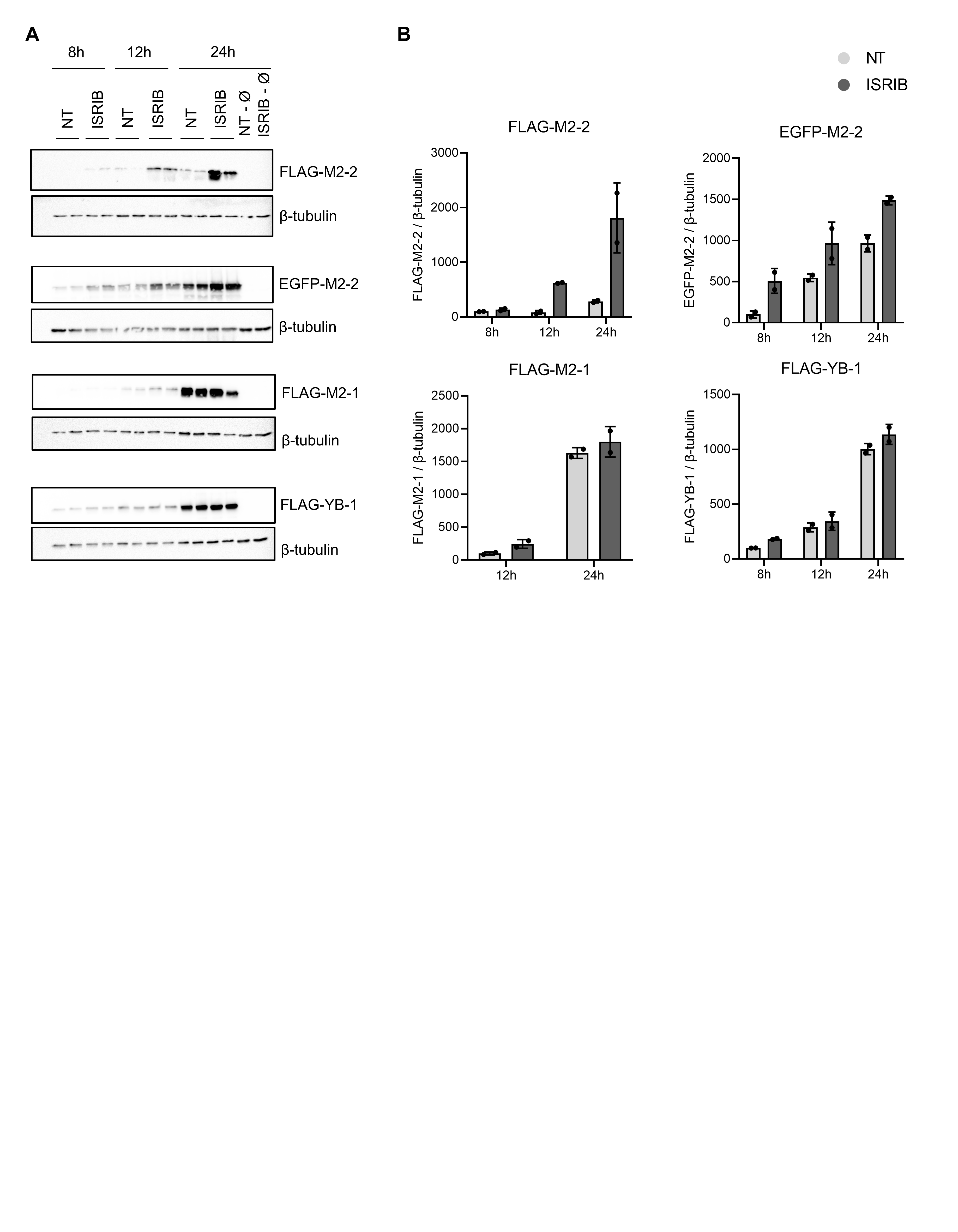

Supplement: S5 Fig — (A) Time-course expression of FLAG-M2-2 and EGFP-M2-2 in HEK293T performed in duplicate. Cells were ISRIB treated (200 nM) or not (NT–non-treated) and collected at indicated times for western blot analysis. As controls, cells were transfected with FLAG-M2-1 or FLAG-YB-1. Quantification of the detected bands is shown in graphs on (B), showing the specific effect of ISRIB in the expression of M2-2. (TIF) [file pone.0289100.s005.TIF]

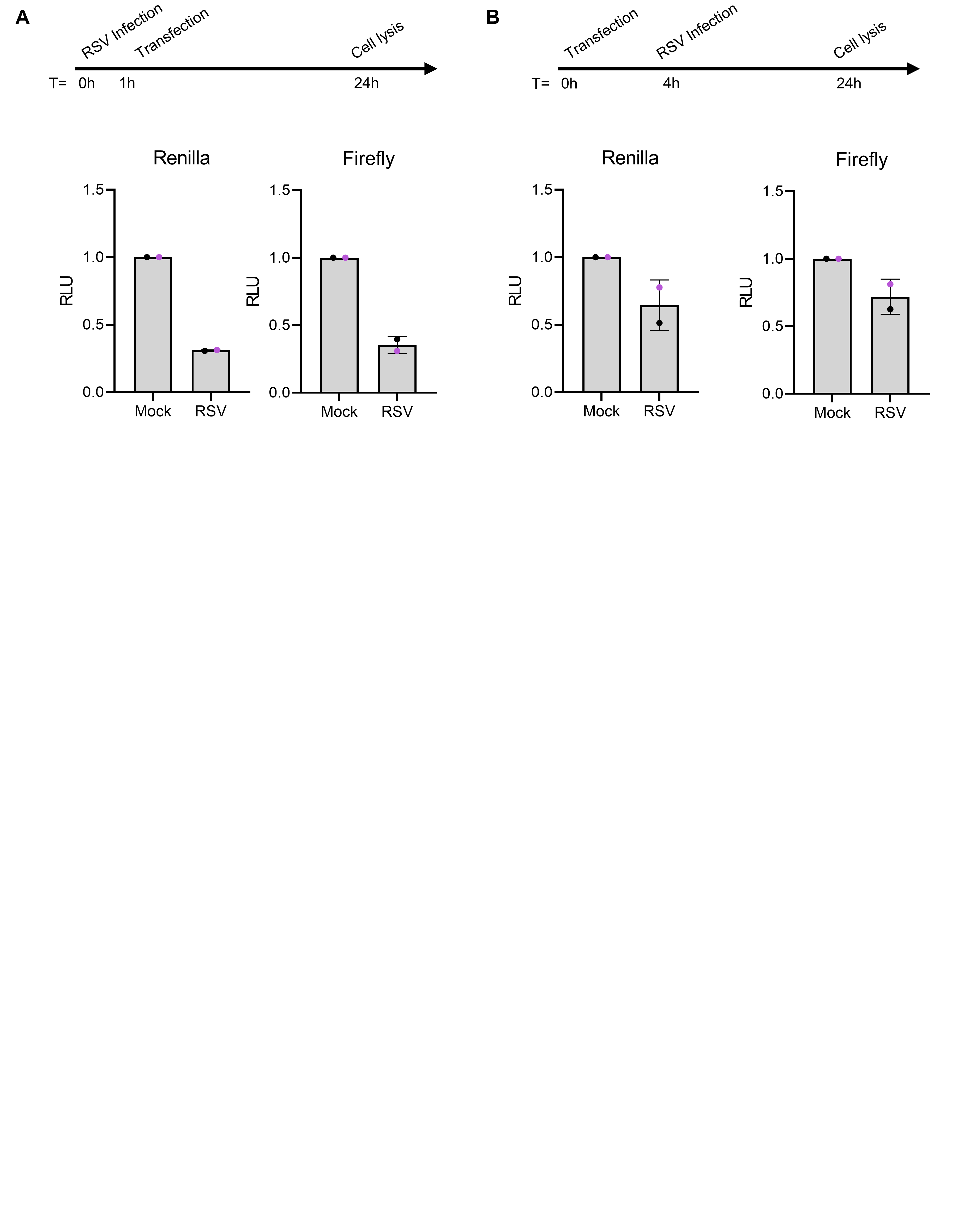

Supplement: S6 Fig — (A) HEK293T cells were mock or RSV infected, and 1 hpi cells were transfected with the vector for expression of reporter luciferases (indicated above). 24 hpi, cells were lysed, and luminescence activity was evaluated, showing inhibition of both Renilla (5’ cap) and Firefly (IRES) luciferases. (B) Alternatively, inhibition was also seen in cells transfected for 4h before infection, following lysis at 20 hpi (as indicated above). Colored dots in the graphs indicate individual means of two independent experiments performed in triplicate (n = 2). Error bars show standard deviation. (TIF) [file pone.0289100.s006.TIF]

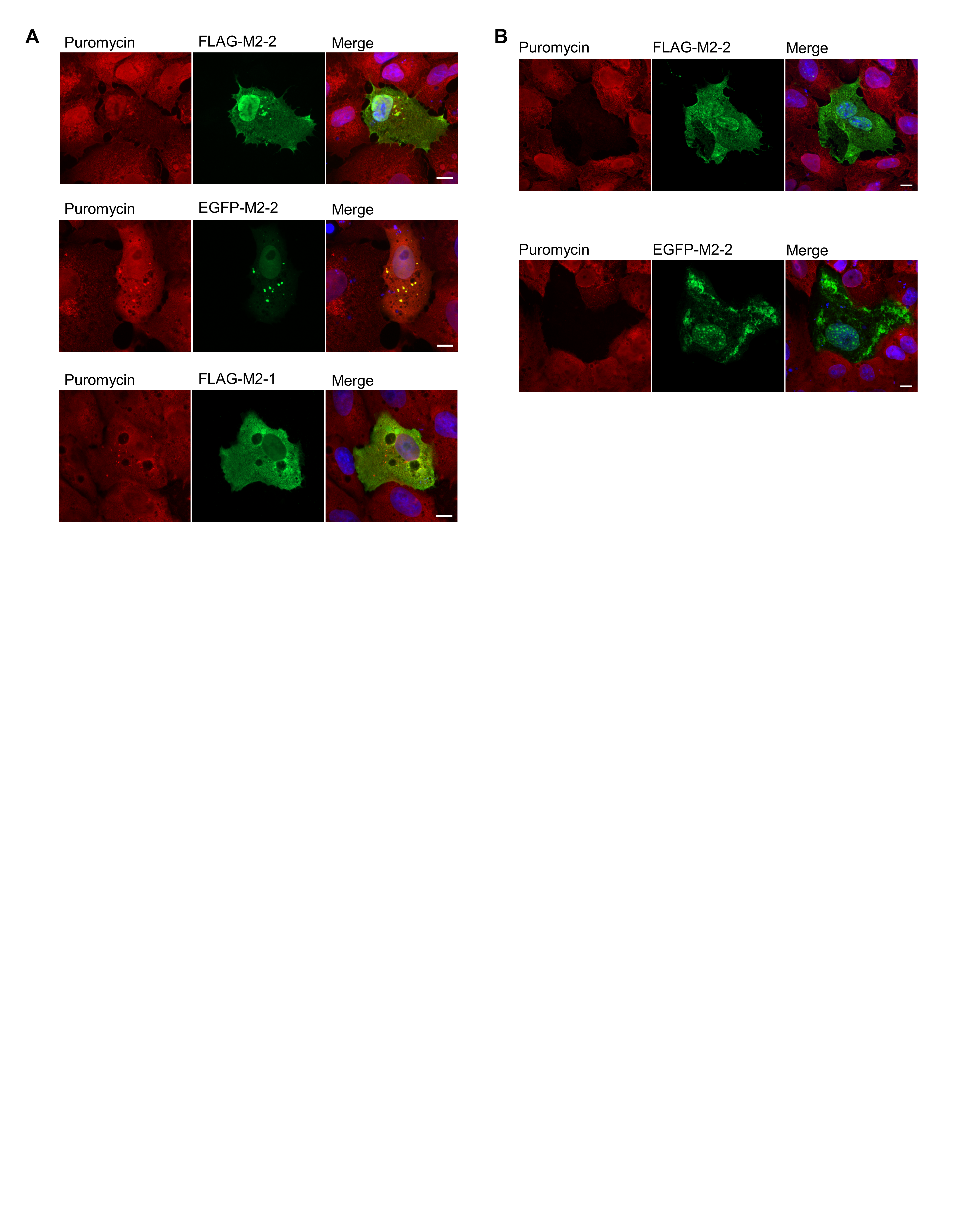

Supplement: S7 Fig — (A) FLAG-M2-2, EGFP-M2-2 and FLAG-M2-1 were expressed in Vero E6 cells and treated with puromycin (10 μg/mL) and MG132 (5 μM) for 2h. Both FLAG and EGFP-M2-2 colocalized with puromycin granules in these cells, with no recruitment of FLAG-M2-1 to puromycin granules. (B) Images show cells were M2-2 expression could inhibit puromycin incorporation, indicating that M2-2 ability to hamper translation is kept in this cell line. All images were taken with a ZEISS Axio Vert.A1 microscope and are representative of two independent experiments. Scale bars 10 μm. (TIF) [file pone.0289100.s007.TIF]

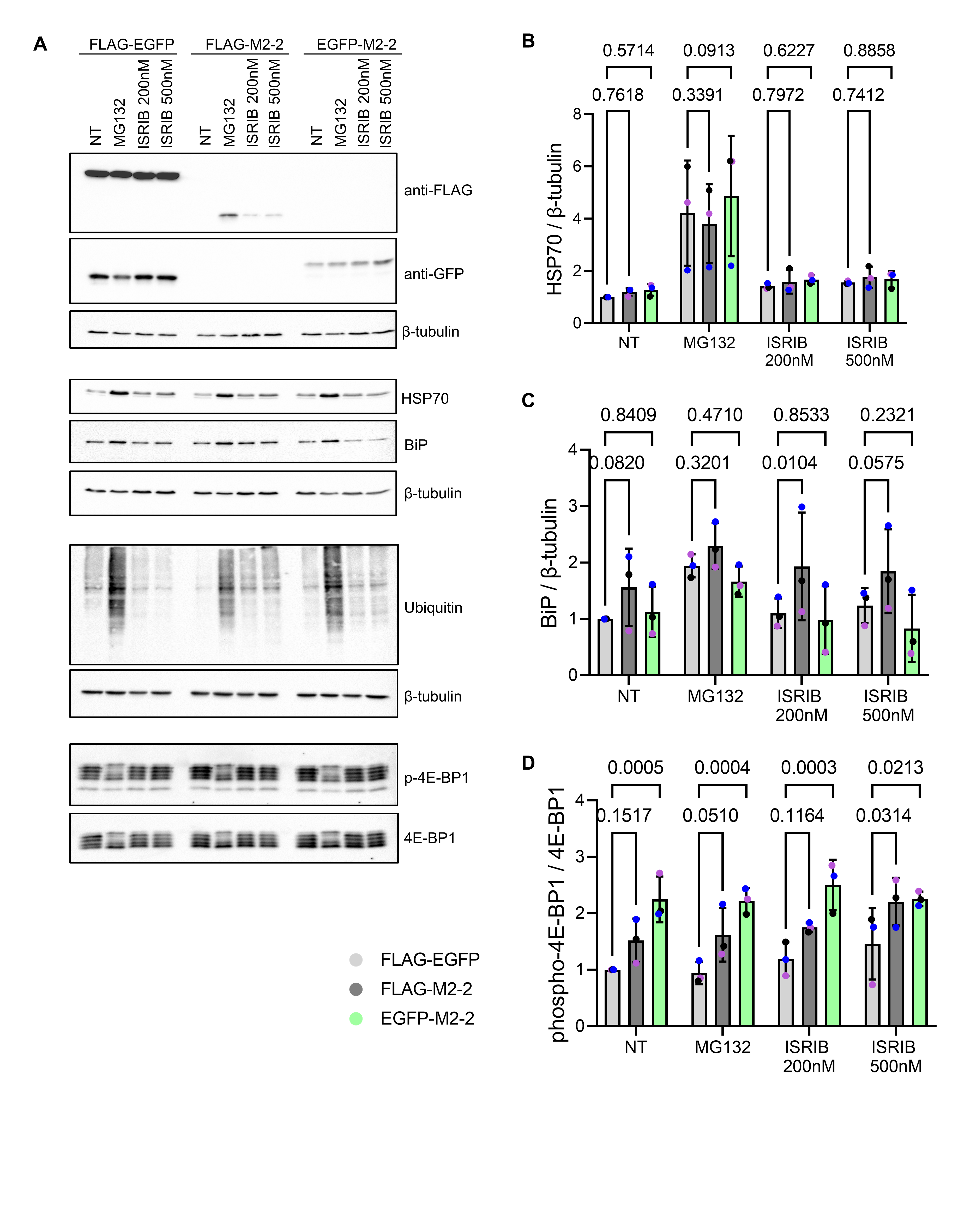

Supplement: S8 Fig — (A) FLAG-EGFP or FLAG and EGFP-M2-2 proteins were expressed in HEK293T under the following treatments, NT–non-treated, MG132 (5 μM), ISRIB (200 nM or 500 nM). After 24h, cells were lysed and extracts were analyzed by western blot, looking for changes in the expression of proteostatic stress markers (HSP70 and BiP), as well as regulators of translation initiation (phospho-4E-BP1). Ubiquitin levels were also accessed for evaluation of proteasome inhibition by MG132. The normalized expression of the proteins is shown as means on the graphs for HSP70 (B), BiP (C) and phosphor-4E-BP1 (D). Statistical differences were evaluated by two-way anova followed by Dunnett’s multiple comparisons test. Colored dots in all graphs indicate in-dividual values from paired independent experiments (n = 3). Error bars indicate standard deviation and p-values are shown in the graphs. (TIF) [file pone.0289100.s008.TIF]

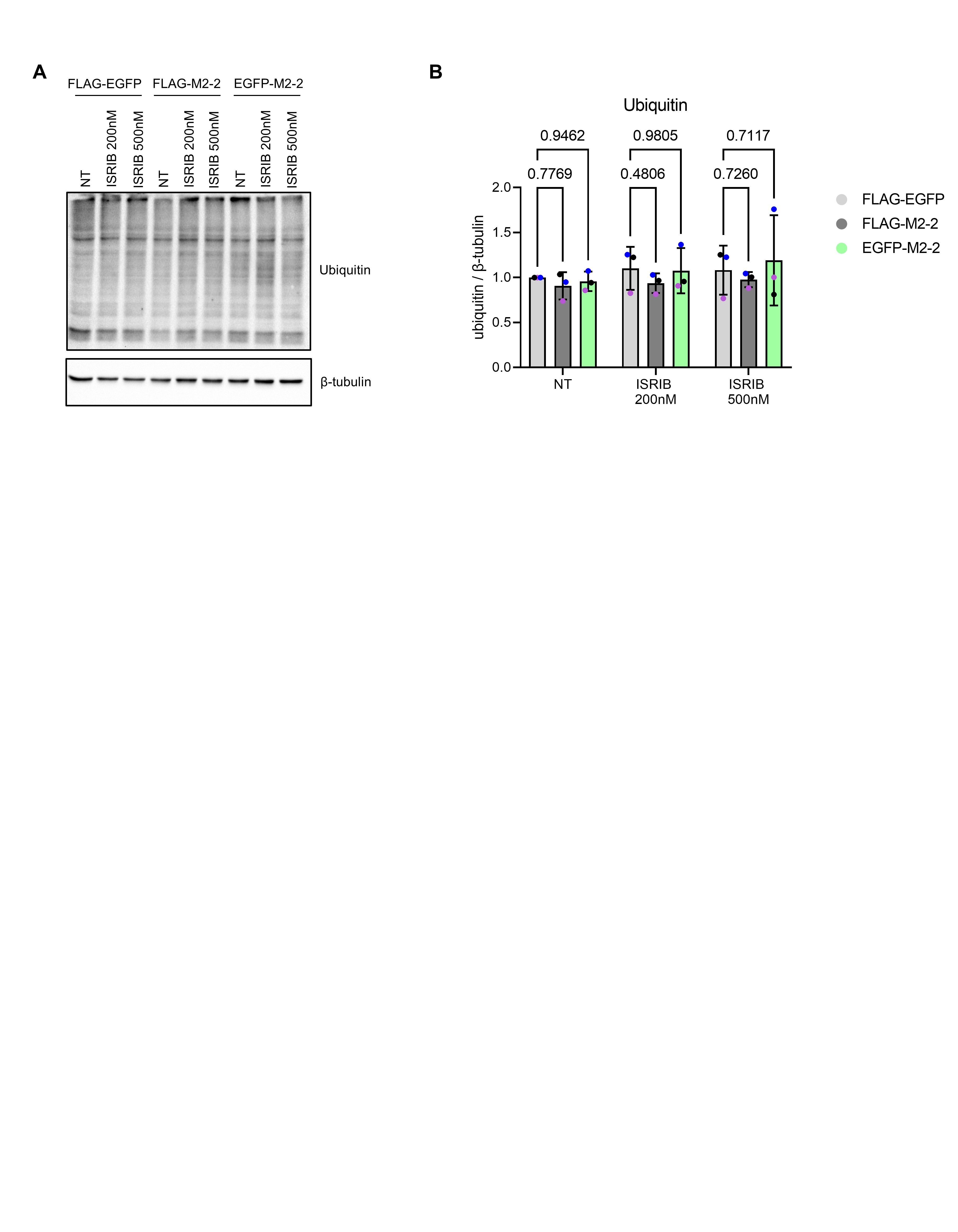

Supplement: S9 Fig — (A) HEK293T cells expressing FLAG-EGFP or FLAG and EGFP-M2-2 proteins were treated or not with ISRIB (as indicated above, NT—non-treated) and had their ubiquitin levels assessed by western blot. Quantitative analysis of three independent experiments is shown as means on the graph in (B), with no significant differences detected between different treatments. Statistical differences were evaluated by two-way anova followed by Dunnett’s multiple comparisons test. Colored dots in graphs indicate individual values from paired independent experiments (n = 3). Error bars indicate standard deviation and p-values are shown in the graphs. (TIF) [file pone.0289100.s009.TIF]
