# Supplementary material for: The respiratory syncytial virus M2-2 protein is targeted for proteasome degradation and inhibits translation and stress granules assembly
Source: PLoS One. 2023 Jul 25;18(7):e0289100. doi: 10.1371/journal.pone.0289100 (PMC10368288; doi:10.1371/journal.pone.0289100)
Supplement: S1 File — (DOCX) [file pone.0289100.s011.docx]

**DNA sequence of optimized genes**

**FLAG-M2-2**

ATGGACTACAAGGACGACGACGACAAGGGCGGCAGCGGCGGAGGCTCTGGCATGACCATGCCCAAGATCATGATTCTGCCCGACAAGTACCCCTGCAGCATCACCAGCATCCTGATCACCAGCCGGTGCAGAGTGACCATGTACAACCAGAAGAACACCCTGTACTTCAACCAGAACAACCCCAACAACCACATGTACAGCCCCAACCAGACCTTCAACGAGATCCACTGGACCAGCCAGGAACTGATCGATACCATCCAGAACTTTCTGCAGCACCTGGGCATCATCGAGGACATCTACACCATCTACATCCTGGTGTCCTGA

**EGFP-M2-2**

ATGGTGTCCAAGGGCGAAGAACTGTTCACCGGCGTGGTGCCCATTCTGGTGGAACTGGACGGGGATGTGAACGGCCACAAGTTTAGCGTTAGCGGCGAAGGCGAAGGGGATGCCACATACGGAAAGCTGACCCTGAAGTTCATCTGCACCACCGGCAAGCTGCCTGTGCCTTGGCCTACACTGGTCACCACACTGACATACGGCGTGCAGTGCTTCAGCAGATACCCCGACCATATGAAGCAGCACGACTTCTTCAAGAGCGCCATGCCTGAGGGCTACGTGCAAGAGCGGACCATCTTCTTTAAGGACGACGGCAACTACAAGACCAGGGCCGAAGTGAAGTTCGAGGGCGACACCCTGGTCAACCGGATCGAGCTGAAGGGCATCGACTTCAAAGAGGACGGCAACATCCTGGGCCACAAGCTCGAGTACAACTACAACAGCCACAACGTGTACATCATGGCCGACAAGCAGAAAAACGGCATCAAAGTGAACTTCAAGATCCGGCACAACATCGAGGACGGCTCTGTGCAGCTGGCCGATCACTACCAGCAGAACACACCCATCGGAGATGGCCCTGTGCTGCTGCCCGATAACCACTACCTGAGCACACAGAGCGCCCTGAGCAAGGACCCCAACGAGAAGAGGGATCACATGGTGCTGCTGGAATTCGTGACCGCCGCTGGCATCACACTCGGCATGGACGAACTGTACAAAGGCGGATCTGGCGGCGGAAGCGGCATGACCATGCCTAAGATCATGATTCTGCCCGACAAGTACCCCTGCAGCATCACCAGCATCCTGATCACCTCCAGATGCAGAGTGACCATGTACAACCAGAAGAACACCCTGTACTTCAACCAGAACAACCCCAACAACCACATGTACAGCCCCAACCAGACCTTCAACGAGATCCACTGGACCAGCCAAGAGCTGATCGATACCATCCAGAACTTTCTGCAGCACCTGGGCATCATCGAGGATATCTACACCATCTACATCCTGGTGTCCTGA

**FLAG-YB-1**

ATGGACTACAAGGACGACGACGACAAAGGCGGCAGCGGCATGTCTAGCGAAGCCGAAACACAGCAGCCTCCAGCTGCTCCTCCTGCAGCTCCTGCTCTTTCTGCCGCCGATACAAAGCCTGGCACAACAGGATCTGGCGCCGGAAGCGGAGGACCTGGTGGACTTACATCTGCTGCTCCAGCCGGCGGAGACAAGAAAGTGATCGCCACAAAGGTGCTGGGCACCGTGAAGTGGTTCAACGTGCGGAATGGCTACGGCTTCATCAACCGGAACGACACCAAAGAAGATGTGTTCGTCCACCAGACCGCCATCAAGAAGAACAACCCCAGAAAGTACCTGCGGAGCGTCGGCGACGGCGAGACAGTGGAATTTGATGTGGTGGAAGGCGAGAAGGGCGCCGAAGCCGCTAATGTTACAGGACCTGGCGGAGTGCCTGTGCAGGGCTCTAAATACGCCGCCGACCGGAACCACTACAGAAGATACCCCAGAAGAAGAGGCCCTCCTCGGAACTACCAGCAGAATTACCAGAACAGCGAGAGCGGCGAGAAGAACGAGGGCTCTGAATCTGCCCCTGAAGGACAGGCTCAGCAGAGAAGGCCTTACAGACGGCGGAGATTCCCTCCATATTACATGCGGAGGCCCTACGGCAGAAGGCCCCAGTACTCTAACCCTCCAGTGCAGGGCGAAGTGATGGAAGGCGCCGATAATCAAGGCGCTGGCGAACAGGGCAGACCCGTGCGGCAGAATATGTACAGAGGCTACAGACCCCGGTTCAGACGGGGACCTCCTAGACAGAGACAGCCCAGAGAGGACGGCAACGAAGAGGACAAAGAGAACCAGGGCGACGAGACACAGGGACAGCAGCCACCTCAGCGGCGGTACAGACGGAACTTCAACTACAGGCGGAGGCGGCCTGAGAACCCCAAACCTCAGGATGGCAAAGAGACAAAGGCCGCCGATCCTCCTGCCGAGAATTCTTCTGCTCCTGAGGCCGAACAAGGCGGAGCCGAATGA

**EGFP-YB-1**

ATGGTGTCCAAGGGCGAAGAACTGTTCACCGGCGTGGTGCCCATTCTGGTGGAACTGGACGGGGATGTGAACGGCCACAAGTTTAGCGTTAGCGGCGAAGGCGAAGGGGATGCCACATACGGAAAGCTGACCCTGAAGTTCATCTGCACCACCGGCAAGCTGCCTGTGCCTTGGCCTACACTGGTCACCACACTGACATACGGCGTGCAGTGCTTCAGCAGATACCCCGACCATATGAAGCAGCACGACTTCTTCAAGAGCGCCATGCCTGAGGGCTACGTGCAAGAGCGGACCATCTTCTTTAAGGACGACGGCAACTACAAGACCAGGGCCGAAGTGAAGTTCGAGGGCGACACCCTGGTCAACCGGATCGAGCTGAAGGGCATCGACTTCAAAGAGGACGGCAACATCCTGGGCCACAAGCTCGAGTACAACTACAACAGCCACAACGTGTACATCATGGCCGACAAGCAGAAAAACGGCATCAAAGTGAACTTCAAGATCCGGCACAACATCGAGGACGGCTCTGTGCAGCTGGCCGATCACTACCAGCAGAACACACCCATCGGAGATGGCCCTGTGCTGCTGCCCGATAACCACTACCTGAGCACACAGAGCGCCCTGAGCAAGGACCCCAACGAGAAGAGGGATCACATGGTGCTGCTGGAATTCGTGACCGCCGCTGGCATCACACTCGGCATGGACGAACTGTACAAAGGCGGATCTGGCGGCGGAAGCGGAATGTCTAGCGAAGCCGAAACACAGCAGCCTCCAGCTGCTCCTCCTGCAGCTCCTGCTCTTTCTGCCGCCGATACAAAGCCTGGCACAACAGGTTCTGGCGCTGGTAGCGGAGGACCTGGTGGACTTACATCTGCTGCTCCAGCCGGCGGAGACAAGAAAGTGATCGCCACAAAGGTGCTGGGCACCGTGAAGTGGTTCAACGTGCGGAATGGCTACGGCTTCATCAACCGGAACGACACCAAAGAGGATGTCTTTGTTCACCAGACCGCCATCAAGAAGAACAACCCCAGAAAGTACCTGCGGAGCGTCGGCGACGGCGAGACAGTGGAATTTGATGTGGTGGAAGGCGAGAAGGGCGCCGAAGCCGCTAATGTTACAGGACCTGGCGGAGTGCCTGTGCAGGGCTCTAAATACGCCGCCGACCGGAACCACTACAGAAGATACCCCAGAAGAAGAGGCCCTCCTCGGAACTATCAGCAGAATTACCAGAACAGCGAGAGCGGCGAGAAGAACGAGGGCTCTGAATCTGCCCCTGAAGGACAGGCTCAGCAGAGAAGGCCTTACAGACGGCGGAGATTCCCTCCATATTACATGCGGAGGCCCTACGGCAGAAGGCCCCAGTACTCTAACCCTCCAGTGCAGGGCGAAGTCATGGAAGGCGCCGATAATCAAGGCGCTGGCGAACAGGGCAGACCCGTGCGGCAGAATATGTACAGAGGCTACAGACCCCGGTTCAGACGGGGACCTCCTAGACAGAGACAGCCCAGAGAGGATGGCAACGAAGAGGACAAAGAGAACCAGGGCGACGAGACACAGGGACAGCAGCCACCTCAGCGGAGATACAGACGGAACTTCAACTACAGGCGGAGGCGGCCTGAGAACCCCAAACCTCAGGACGGAAAAGAGACAAAGGCCGCCGATCCTCCTGCCGAGAATTCTTCTGCTCCTGAGGCCGAACAAGGCGGAGCCGAATGA

**M2-1**

ATGAGCCGGCGGAACCCCTGCAAGTTCGAGATCCGGGGCCACTGCCTGAACGGCAAGCGGTGCCACTTCAGCCACAACTACTTCGAGTGGCCCCCTCACGCCCTGCTGGTCCGCCAGAACTTCATGCTGAACAGAATCCTGAAGTCCATGGACAAGAGCATCGACACCCTGAGCGAGATCAGCGGAGCCGCCGAGCTGGACCGGACCGAGGAATATGCCCTGGGCGTGGTCGGAGTGCTGGAAAGCTACATCGGCAGCATCAACAACATCACCAAGCAGAGCGCCTGCGTGGCCATGAGCAAGCTGCTGACCGAGCTGAACAGCGACGACATCAAGAAGCTGCGGGACAACGAGGAACTGAACAGCCCCAAGATCCGGGTGTACAACACCGTGATCAGCTACATCGAGAGCAACCGGAAGAACAACAAGCAGACCATCCATCTGCTGAAGCGGCTGCCCGCCGACGTGCTGAAGAAAACCATCAAGAACACCCTGGACATCCACAAGTCCATCACCATCAACAACCCCAAAGAAAGCACCGTGTCCGACACCAACGACCACGCCAAGAACAACGACACCACCTGA
