# Supplementary material for: The respiratory syncytial virus M2-2 protein is targeted for proteasome degradation and inhibits translation and stress granules assembly
Source: PLoS One. 2023 Jul 25;18(7):e0289100. doi: 10.1371/journal.pone.0289100 (PMC10368288; doi:10.1371/journal.pone.0289100)

Figure 1 - A

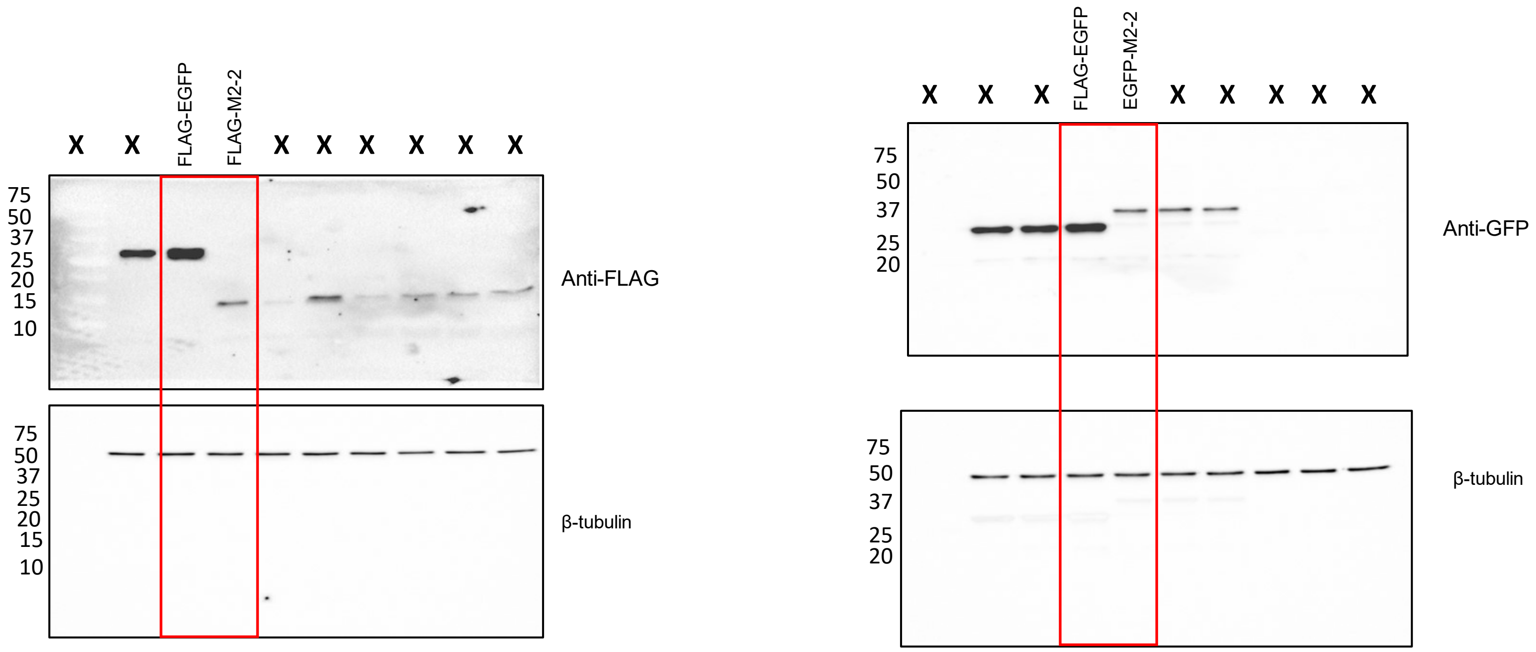
X X FLAG-EGFP FLAG-M2-2 X X X X X X
75 50 37 25 20 15 10
Anti-FLAG
75 50 37 25 20 15 10
β-tubulin
X X X FLAG-EGFP EGFP-M2-2 X X X X X
75 50 37 25 20
Anti-GFP
75 50 37 25 20
β-tubulin

Figure 3 - A

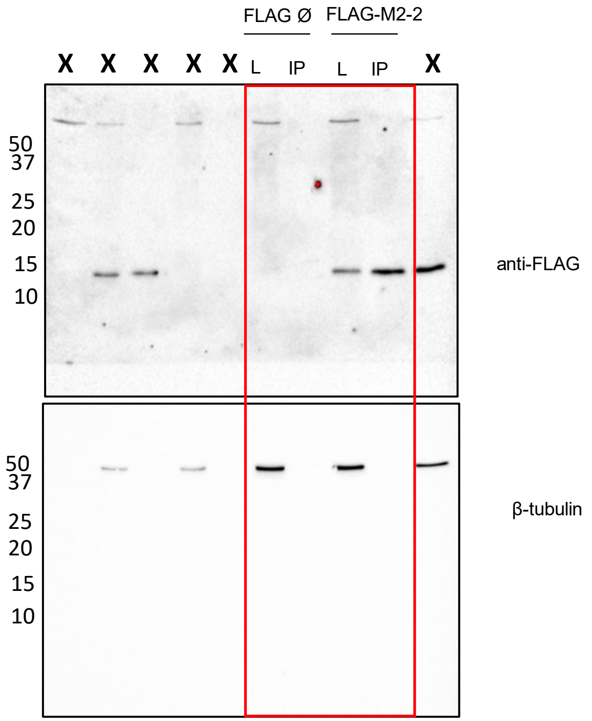
FLAG Ø
FLAG-M2-2
X X X X X L IP L IP X
50
37
25
20
15
10
anti-FLAG
50
37
25
20
15
10
β-tubulin

Figure 5 - C

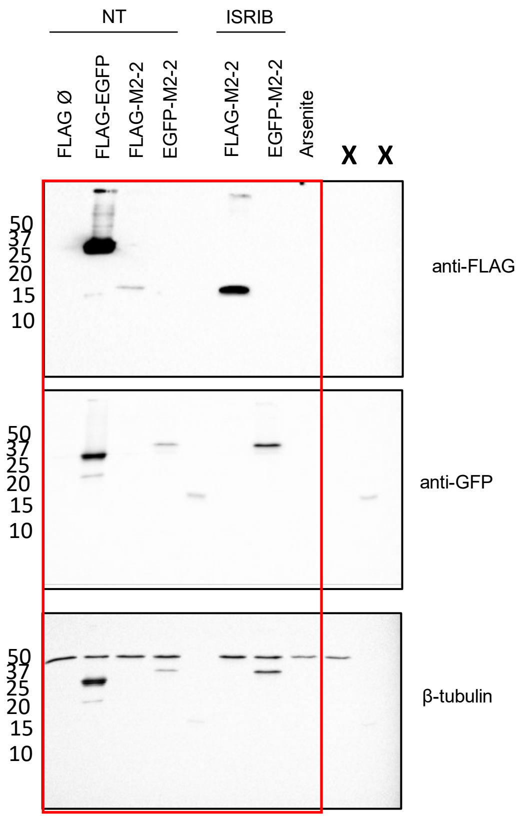
NT
ISRIB
FLAG Ø
FLAG-EGFP
FLAG-M2-2
EGFP-M2-2
FLAG-M2-2
EGFP-M2-2
Arsenite
X
X
50
37
25
20
15
10
anti-FLAG
50
37
25
20
15
10
anti-GFP
50
37
25
20
15
10
β-tubulin

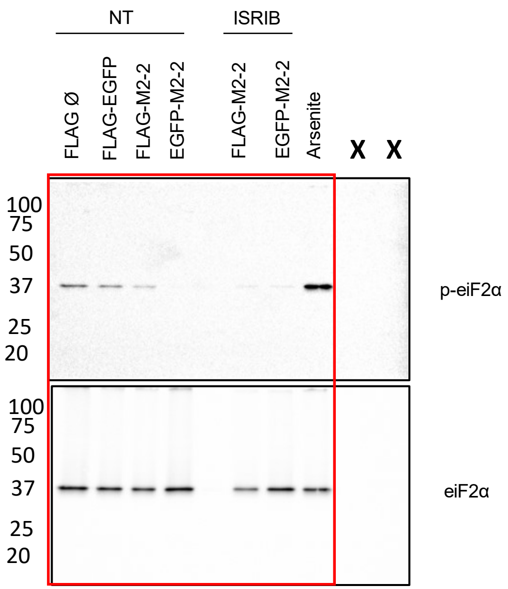
NT
ISRIB
FLAG Ø
FLAG-EGFP
FLAG-M2-2
EGFP-M2-2
FLAG-M2-2
EGFP-M2-2
Arsenite
X
X
100
75
50
37
25
20
p-eiF2α
100
75
50
37
25
20
eiF2α

Figure 6 - A

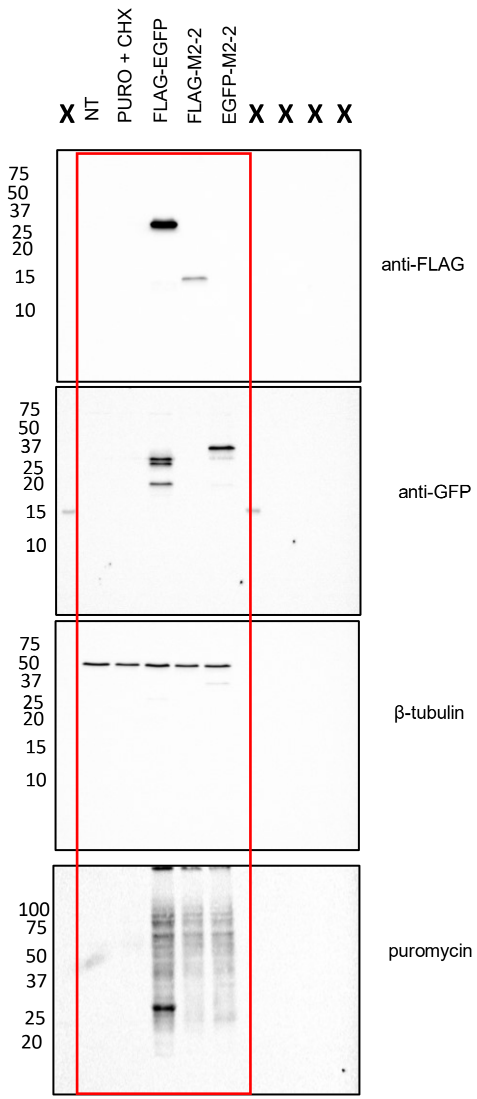
X
NT
PURO + CHX
FLAG-EGFP
FLAG-M2-2
EGFP-M2-2
X X X X
75
50
37
25
20
15
10
anti-FLAG
75
50
37
25
20
15
10
anti-GFP
75
50
37
25
20
15
10
β-tubulin
100
75
50
37
25
20
puromycin

Figure 7 - A

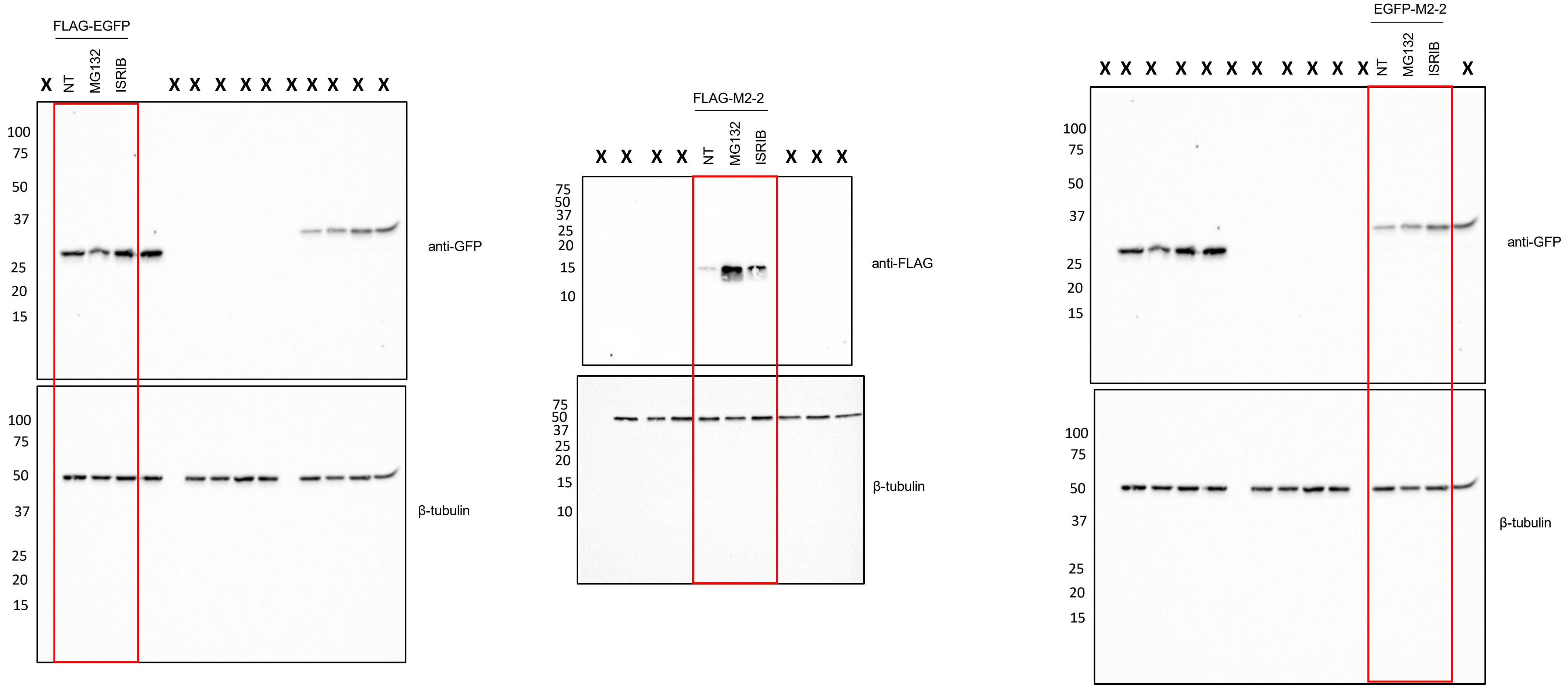
FLAG-EGFP
X NT MG132 ISRIB X X X X X X X X X X
100
75
50
37
25
20
15
anti-GFP
100
75
50
37
25
20
15
β-tubulin
FLAG-M2-2
X X X X NT MG132 ISRIB X X X
75
50
37
25
20
15
10
anti-FLAG
75
50
37
25
20
15
10
β-tubulin
EGFP-M2-2
X X X X X X X X X X X NT MG132 ISRIB X
100
75
50
37
25
20
15
anti-GFP
100
75
50
37
25
20
15
β-tubulin

Figure 7 - C

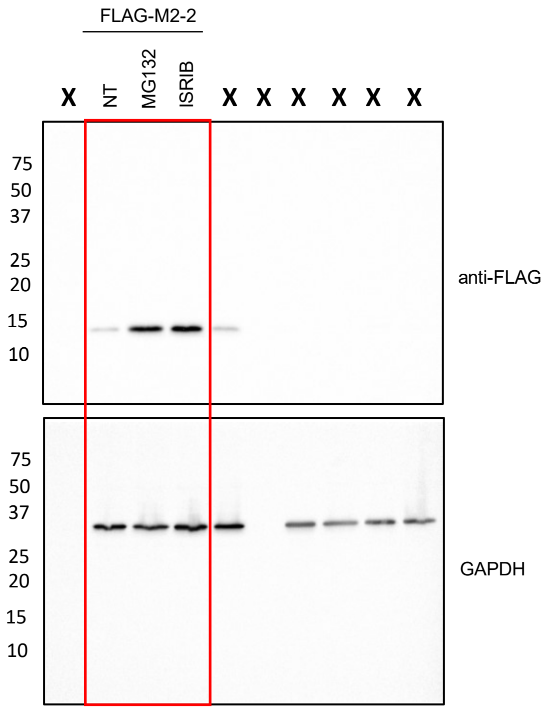
FLAG-M2-2
X
NT
MG132
ISRIB
X
X
X
X
X
X
75
50
37
25
20
15
10
anti-FLAG
75
50
37
25
20
15
10
GAPDH

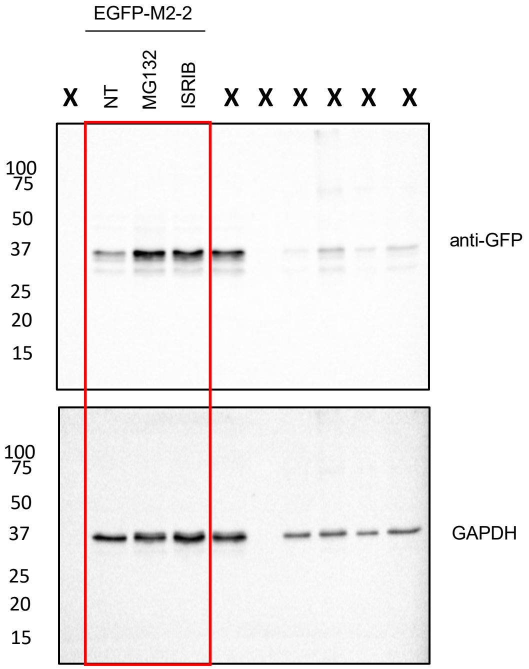
EGFP-M2-2
X
NT
MG132
ISRIB
X
X
X
X
X
X
100
75
50
37
25
20
15
anti-GFP
100
75
50
37
25
20
15
GAPDH

Figure 7 - D

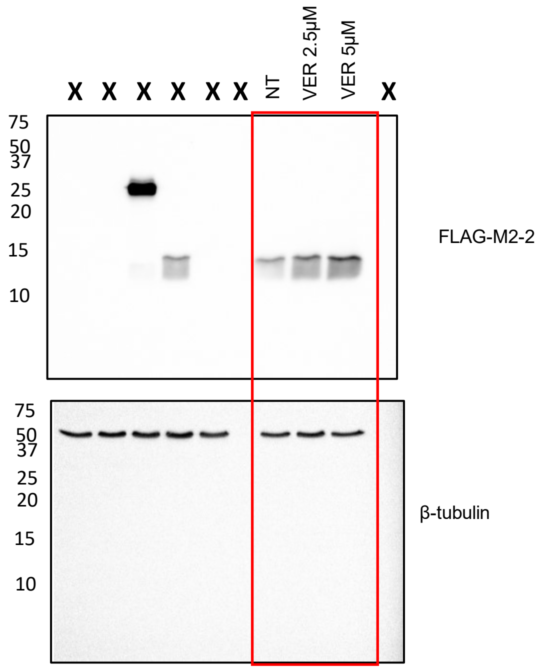
X X X X X X NT VER 2.5μM VER 5μM X
75
50
37
25
20
15
10
FLAG-M2-2
75
50
37
25
20
15
10
β-tubulin

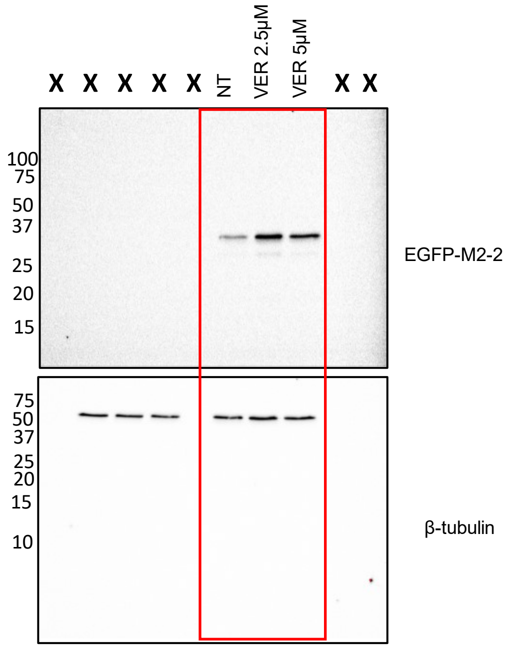
X X X X X NT VER 2.5μM VER 5μM X X
100
75
50
37
25
20
15
EGFP-M2-2
75
50
37
25
20
15
10
β-tubulin

Figure 10 – A, B and C

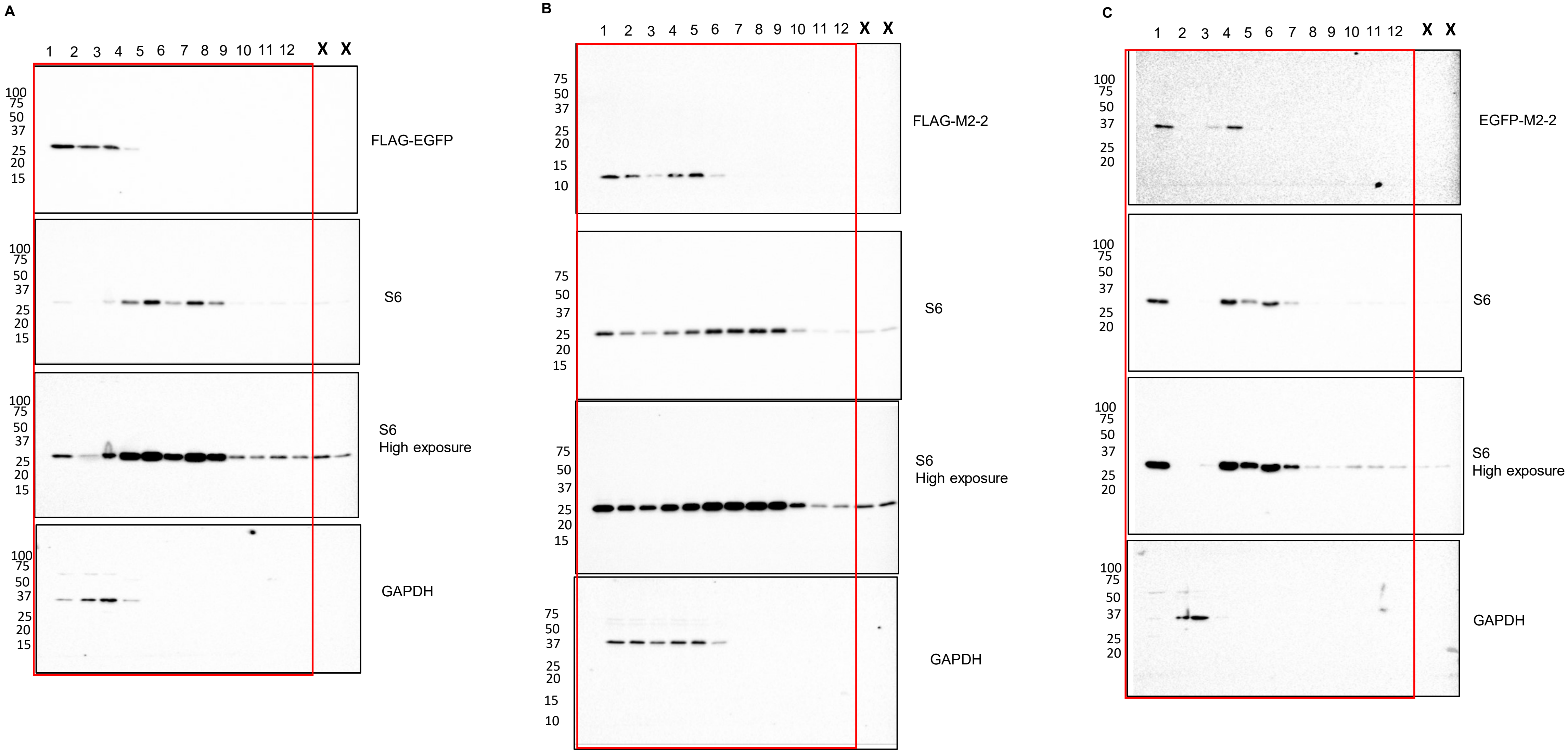

Supplementary Figure 4 - A

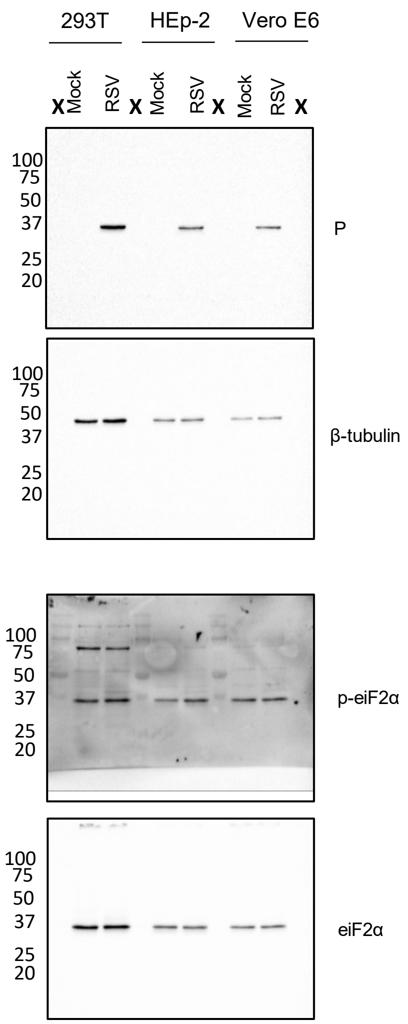

Supplementary Figure 5 - A

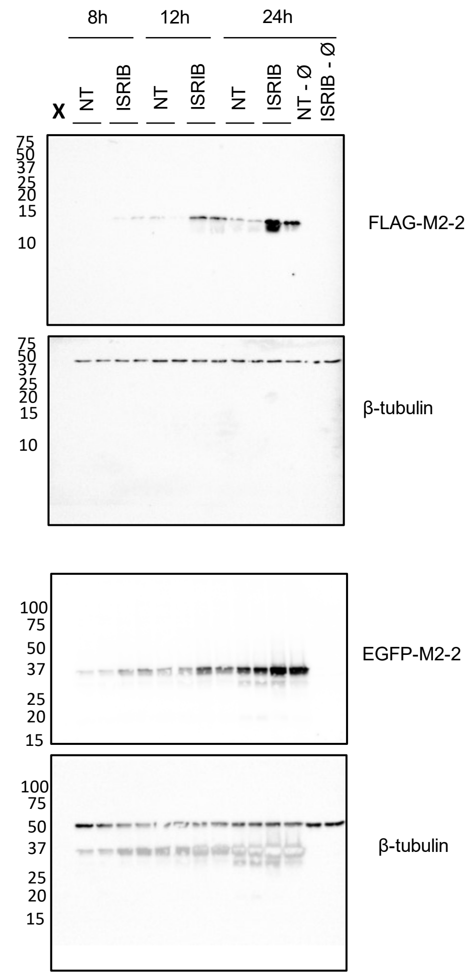

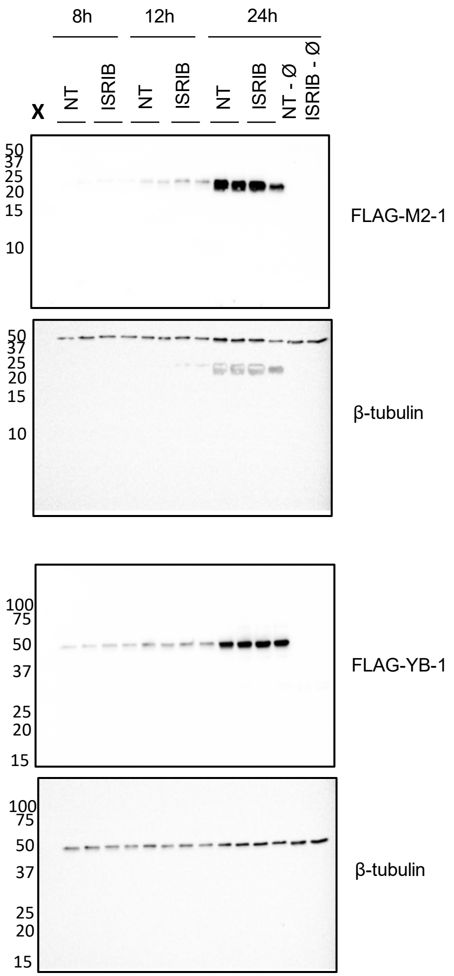

Supplementary Figure 8 - A

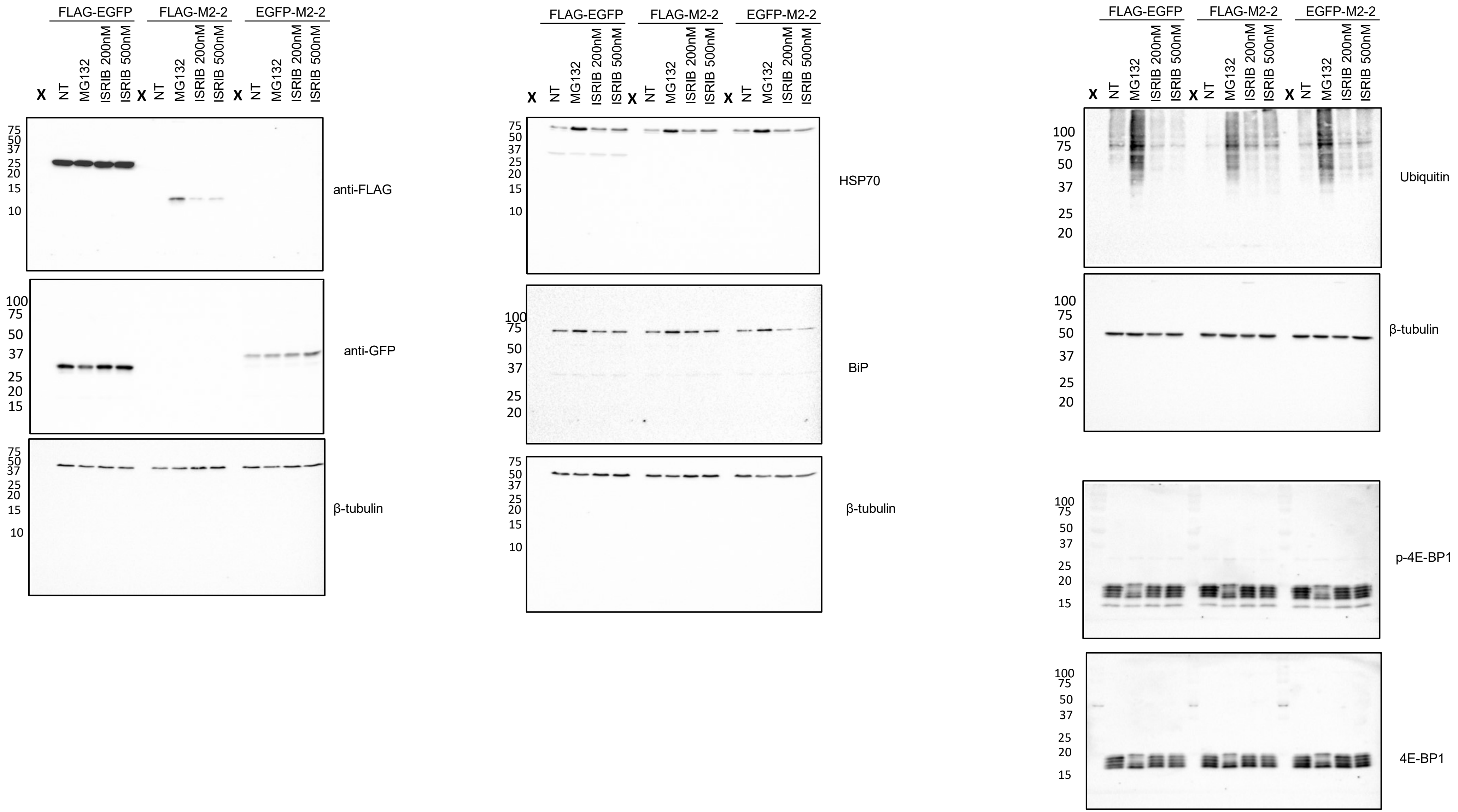

Supplementary Figure 9 - A

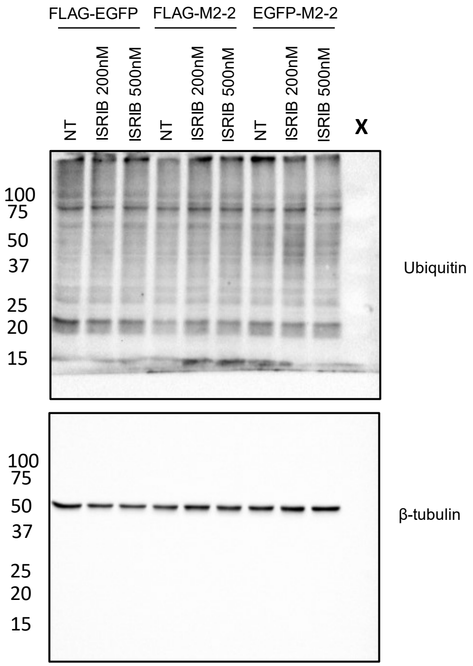

Supplement: S1 Raw images — (PDF) [file pone.0289100.s012.pdf]
